# The prediction of interferon treatment effects based on time series microarray gene expression profiles

**DOI:** 10.1186/1479-5876-6-44

**Published:** 2008-08-09

**Authors:** Tao Huang, Kang Tu, Yu Shyr, Chao-Chun Wei, Lu Xie, Yi-Xue Li

**Affiliations:** 1 Key Laboratory of Systems Biology, Shanghai Institutes for Biological Sciences, Chinese Academy of Sciences, Shanghai, 200031, PR China; 2 Shanghai Center for Bioinformation Technology, Shanghai, 200235, PR China; 3 Cancer Biostatistics Center, Vanderbilt University, Nashville, 37232, USA

## Abstract

**Background:**

The status of a disease can be reflected by specific transcriptional profiles resulting from the induction or repression activity of a number of genes. Here, we proposed a time-dependent diagnostic model to predict the treatment effects of interferon and ribavirin to HCV infected patients by using time series microarray gene expression profiles of a published study.

**Methods:**

In the published study, 33 African-American (AA) and 36 Caucasian American (CA) patients with chronic HCV genotype 1 infection received pegylated interferon and ribavirin therapy for 28 days. HG-U133A GeneChip containing 22283 probes was used to analyze the global gene expression in peripheral blood mononuclear cells (PBMC) of all the patients on day 0 (pretreatment), 1, 2, 7, 14, and 28. According to the decrease of HCV RNA levels on day 28, two categories of responses were defined: good and poor. A voting method based on Student's t test, Wilcoxon test, empirical Bayes test and significance analysis of microarray was used to identify differentially expressed genes. A time-dependent diagnostic model based on C4.5 decision tree was constructed to predict the treatment outcome. This model not only utilized the gene expression profiles before the treatment, but also during the treatment. Leave-one-out cross validation was used to evaluate the performance of the model.

**Results:**

The model could correctly predict all Caucasian American patients' treatment effects at very early time point. The prediction accuracy of African-American patients achieved 85.7%. In addition, thirty potential biomarkers which may play important roles in response to interferon and ribavirin were identified.

**Conclusion:**

Our method provides a way of using time series gene expression profiling to predict the treatment effect of pegylated interferon and ribavirin therapy on HCV infected patients. Similar experimental and bioinformatical strategies may be used to improve treatment decisions for other chronic diseases.

## Background

Chronic diseases such as infectious disease, cancer, and diabetes are among the most common and costly health problems. The therapy of chronic diseases often lasts for a long time, while the treatment effect may be questionable and yet the side effects may be serious. Hepatitis C virus (HCV) is one of the major causes of chronic hepatitis, cirrhosis, and hepatocellular carcinoma. The current recommended treatment for chronic HCV infection is the combination of pegylated alpha interferon (peginterferon) and the oral antiviral drug ribavirin given for 24 or 48 weeks, but the chance to induce a sustained response is only 54%–56%[1]. Using interferon and ribavirin for a long time may cause serious side effects, such as fever, chills, body aches, headaches, myeloid disorders[2] and neuropsychiatric symptoms[3]. The patients with poor response should better give up such treatment in the early stage. However the underlying mechanisms for different responses are not fully understood and it is hard to foresee treatment effects by conventional methods.

We analyzed a published time series microarray dataset of a virological research in which the effects of pegylated interferon and ribavirin on 33 African-American (AA) and 36 Caucasian American (CA) patients with chronic HCV infection were studied[4]. We established a diagnostic model to predict the outcome of pegylated interferon and ribavirin therapy using time series microarray gene expression profiles for AA and CA patients separately.

Although the focus here is on how HCV infected patients respond to pegylated interferon treatment, the model described is generally applicable to other chronic diseases undergoing long term treatment.

## Methods

### Original time-series microarray data applied in our study

The original time-series microarray data used in this work is from a study of Milton W. Taylor which was published on Journal of Virology last year[4], and publicly available at GEO  under accession number GSE7123. The initial data set consists of the gene expression profiles of 33 African-American and 36 Caucasian American patients with chronic HCV genotype 1 infection on day 0 (pretreatment), and 1, 2, 7, 14, and 28 of pegylated interferon and ribavirin therapy. HG-U133A GeneChip containing 22283 probes was used to analyze the global gene expression in peripheral blood mononuclear cells (PBMC) of the patients at each time point. For each patient the decrease of HCV RNA level was calculated by subtracting baseline level (before treatment) from the level on day 28. Good response was defined as a decrease of more than 1.4 log_10 _IU/ml of HCV RNA level; and poor response was defined as less than 1.4 log_10 _IU/ml decline from the base level. Only patients with all the gene expression data of 6 time points were involved in our analysis, including 30 Caucasian Americans (CA) of whom 17 were good responders and 13 were poor, 28 African-Americans (AA) of whom 19 were good responders and 9 were poor.

### Data preprocessing

First, we normalized the data of total 348 microarrays using quantile method and log_2_-trasformed them. Only probes that were present in at least 75% microarrays with log_2 _intensities greater than 7 were kept for further analysis. This resulted in a subset of approximately 13620 probesets representing 9100 different genes.

### Statistical analysis to identify differentially expressed genes

To avoid bias that may be created by single feature-selecting statistical method, we constructed a voting method based on several methods including Student's t test[5], Wilcoxon test[6], empirical Bayes test (eBayes) [7]and significance analysis of microarray(SAM)[8] to identify differentially expressed genes. Only genes that passed three out of the four methods were regarded as differentially expressed. The selection criterion was set at a defined P value for all four statistical tests.

### Time-dependent diagnostic model

**Figure 1:**
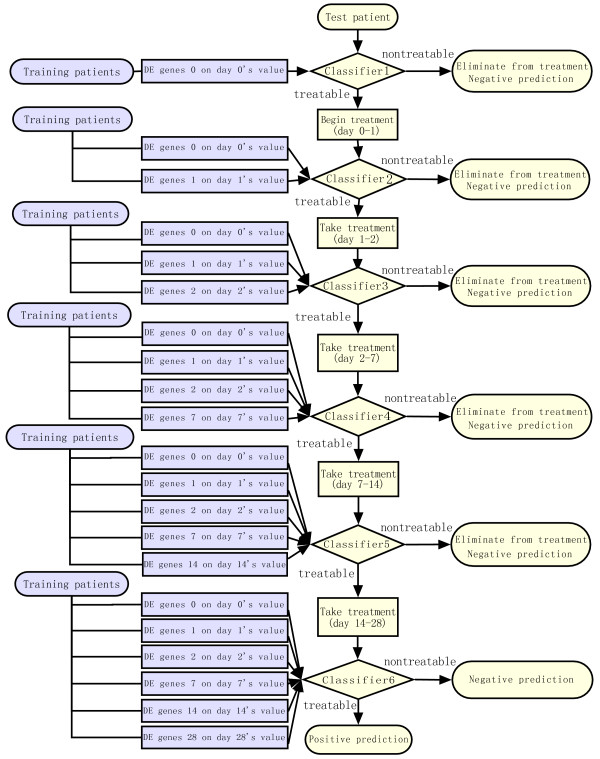
**The framework of time-dependent diagnostic model**. "nontreatable" means the patient was predicted to have a poor response and should be eliminated from the treatment, "treatable" means the patient was predicted to have a good response and should keep the treatment.

The classifier used in our program at each time point was C4.5-a decision tree classification method [9]. With leave-one-out cross-validation, the model was trained and tested at each time point. The framework is illustrated in Figure 1 and detailed as follows:

#### Train the model

Each patient in the training set was regarded as an instance and the class label for him was the outcome of the treatment. At each time point, differentially expressed genes between good and poor response group were identified using the voting method described above as the marker probe sets of this time point. At the first time point (time point 0, before treatment), the features were that day's gene expression values of the marker probe sets at that time point; at the following time point during the treatment, the features were the combination of that day's gene expression values of marker probe sets at that time point and features of previous time points. For example, the features at day 1 are the expression values of differentially expressed genes at day 1 and the expression values of differentially expressed genes at day 0.

Every patient is assumed treatable until predicted as nontreatable with sufficient differentially expressed genes at that day. For each time point, if the number of differentially expressed genes was equal or greater than 5, the C4.5 classifier will be constructed at this time point; otherwise, differentially expressed gene number at this time point will be set as null and no C4.5 classifier will be trained at this time point. This check step helps to avoid false negative decision.

#### Test the model

At each time of leave-one-out cross-validation, we used the data of N-1 patients to build a model and then applied it on the one left patient to predict his treatment outcome. If a patient was predicted as treatable by every time point's classifier, this was a positive prediction. If the final outcome according to the HCV RNA level decline was good response for this patient, this was a true positive prediction; otherwise, it was a false positive prediction.

If a patient was predicted as nontreatable by one of the six classifiers (day 0, 1, 2, 7, 14 and 28), this was a negative prediction. That means this patient should be eliminated from the treatment and the workflow of this patient will stop at that time point. If the real outcome was poor response for this patient, this was a true negative prediction; otherwise, this was a false negative prediction.

The prediction accuracy Q of leave-one-out cross-validation was calculated as follows:

$$Q=\frac{tp+tn}{tp+tn+fp+fn}\times 100$$

tp, tn, fp and fn stand for true positive, true negative, false positive and false negative, respectively. Detailed information about this model, including processed microarray data, R code and results, can be found in Additional file 1 and file 2.

### Relevance and significance of candidate biomarkers

To assess the biological relevance of the identified candidate biomarkers which were important for CA response prediction, we used PubGene to find relationships between these candidate biomarkers and IFN (Interferon)/HCV (Hepatitis C viruses). PubGene  is a tool to carry out automated extraction of explicit and implicit biomedical knowledge from publicly available gene and text databases to create a gene-to-gene co-citation network by automated analysis of titles and abstracts in MEDLINE records[10]. Moreover, GO and KEGG category enrichment analyses were applied to validate the functional and pathway relevance of selected candidate biomarkers.

## Results

### Time-dependent diagnostic model

The true power of time series microarray analysis does not come from the analysis of single time point, but rather, from the analysis of a series of time points to identify a biomarker chain. The main idea of our model is to fully utilize gene expression profiles before and during treatment to predict the final treatment outcome.

**Figure 2:**
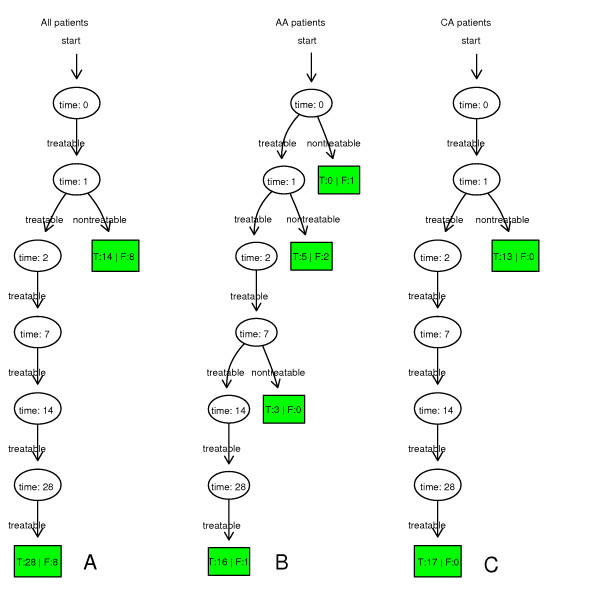
**The leave-one-out cross-validation results of time-dependent diagnostic models based on all patients, AA patients and CA patients**. On day one, most nontreatable patients could be detected correctly. "T" means true prediction; "F" means false prediction.

The time-dependent diagnostic results of all patients, AA patients and CA patients are shown in Figure 2. It illustrates that true negative CA patients were all correctly detected on day 1 and true negative AA patients were mostly detected on day 1 and 7. The leave-one-out cross-validation results of all patients, AA patients and CA patients are given in Table 1. In the preliminary research, we found that the numbers of differentially expressed genes in all patients, AA patients and CA patients under the same cut-off P value were quite different. To balance the numbers of differentially expressed genes in different groups, P values given to identify differentially expressed genes in all patients, AA patients and CA patients were 0.00001, 0.001 and 0.0001, respectively.

**Table 1:** The Leave-one-out cross-validation results of all patients, AA patients and CA patients.

	All patients			AA patients			CA patients		
		Predicted Good	Predicted poor		Predicted Good	Predicted poor		Predicted Good	Predicted poor

2 × 2 Table	Actual Good	28	8	Actual Good	16	3	Actual Good	17	0
	Actual poor	8	14	Actual poor	1	8	Actual poor	0	13

Accuracy		72.4%			85.7%			100%	

* Good means good response, poor means poor response.

### Simplified Time-dependent diagnostic model

We have known that if only static gene expression profiles before treatment were used the prediction accuracy was rather low (data not shown). However from the above results, it occurred to us that the seemingly complicated models may actually be simplified to day 1 classifier – depending only on gene expression profiles of very early treatment time point. The leave-one-out cross-validation accuracy based on day 1 classifier (including day 0 and day 1 gene expression profiles) of CA patients could achieve 100%, the same as the result using data of all the time points. With AA patients, the accuracy dropped some, but still much better than if only using pre-treatment gene expression profile. The leave-one-out cross-validation results of all patients, AA patients and CA patients on day 1 are given in Table 2.

**Table 2:** The leave-one-out cross-validation results of all patients, AA patients and CA patients on day 1.

	All patients			AA patients			CA patients		
		Predicted Good	Predicted poor		Predicted Good	Predicted poor		Predicted Good	Predicted Poor

2 × 2 Table	Actual Good	28	8	Actual Good	16	3	Actual Good	17	0
	Actual poor	8	14	Actual poor	4	5	Actual poor	0	13

Accuracy		72.4%			75%			100%	

* Good means good response, poor means poor response.

### Identification of candidate biomarkers of CA patients

As stated above, CA patients of HCV infection are more sensitive to the therapy of interferon and ribavirin, and after one day treatment the outcome could be one hundred percent predicted. Using the feature selection methods described in Methods section, we identified 30 differentially expressed genes or probes on day one between 17 good response CA patients and 13 poor response CA patients as the candidate biomarkers relevant to interferon therapy response. They are EIF3S5, HSPA9, ABLIM1, RPL4 (201154_x_at), MARCKS, HTRA2, SH2B3, KIAA0999, LCK, C8orf70, TTLL1, CD86, TUFT1, KLRK1, PARP1, KPNB1, NT5C2, RPL4 (211710_x_at), MRPS27, AOF2, HSD17B8, RBMX, TNFSF10, SMARCA4, C14orf122, KIAA0748, PCID2, DNAPTP6, TLE2 and CYFIP2. Their detailed probe information are provided in Additional file 3.

**Figure 3:**
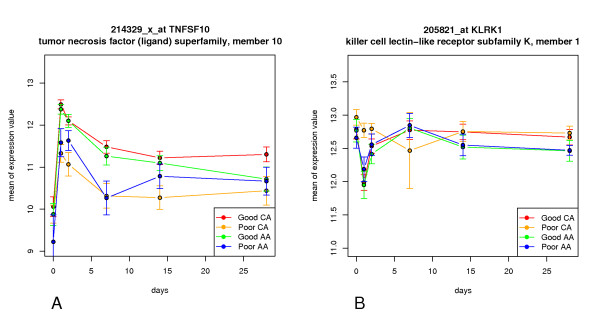
**The time series expression of two representative genes TNFSF10 (tumor necrosis factor (ligand) superfamily, member 10) and KLRK1 (killer cell lectin-like receptor subfamily K, member 1)**. The horizontal axis depicts days that the treatment has lasted, the vertical axis stands for expression mean values with error bar. Different colour indicates different groups of patients.

The time series expression profiles of two representative genes TNFSF10 (tumor necrosis factor (ligand) superfamily, member 10) and KLRK1 (killer cell lectin-like receptor subfamily K, member 1) are shown in Figure 3. In Figure 3A, at most of the time, the difference of TNFSF10 expression level between Good response CA and Poor response CA is much greater than the difference between Good response AA and Poor response AA. Moreover, the difference between Good response CA and Poor response CA increases with time, but the difference between Good response AA and Poor response AA decreases with time. In Figure 3B, the same phenomenon can be observed on day 1 and 7. Many other genes showed similar tendency at earlier time points. Maybe this partly explains why the treatment of CA patients can be more accurately predicted than AA patients. The dynamic expression graphics of all thirty genes are provided in Additional file 4.

**Figure 4:**
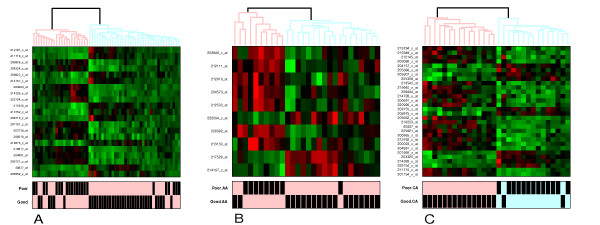
Unsupervised two-way hierarchical clustering based on expression profiles of differentially expressed genes of all patients on day 1(A), AA patients on day 1(B), and CA patients on day 1(C). P values given to identify differentially expressed genes in all patients, AA patients and CA patients were 0.00001, 0.001 and 0.0001, respectively. The bar indicates the response status of patients.

To further evaluate whether these expression signatures are associated with therapeutic outcome (good or poor response), we conducted clustering of CA patients using differentially expressed genes on day 1 between CA groups of good and poor outcome (Figure 4C), and compared it with the clustering result of all patients using differentially expressed genes on day 1 between all patients of good and poor outcome(Figure 4A), of AA patients using differentially expressed genes on day 1 between AA groups of good and poor outcome (Figure 4B).

It can be seen that Figure 4C best clustered its patients. These thirty genes could classify the CA patients into good responders and poor responders very well. Therefore the simplified day 1 diagnostic model can clearly be applied to CA patients.

### Relevance and significance of candidate biomarkers

The relationship between those candidate biomarkers and IFN (Interferon)/HCV (Hepatitis C viruses) were explored by using PubGene. The two literature networks are shown in Additional file 5, indicating six genes that have direct connections with both IFN and HCV. They are LCK (lymphocyte-specific protein tyrosine kinase), NT5C2 (5'-nucleotidase, cytosolic II), KLRK1 (killer cell lectin-like receptor subfamily K, member 1), CD86 (CD86 antigen (CD28 antigen ligand 2, B7-2 antigen)), PARP1 (poly (ADP-ribose) polymerase family, member 1) and TNFSF10 (tumor necrosis factor (ligand) superfamily, member 10). It has been reported that IFN-alpha can significantly enhance CD86 expression on dendritic cells from chronic hepatitis C patients[11,12]. The ligation of tumor necrosis factor receptor (TNFR1) can initiate apoptosis or programmed cell death which is part of interferon (IFN)-mediated anti-viral action[13].

GO category enrichment analysis results (see Additional file 6) show that many of these candidate biomarkers are involved in immunity, such as T cell differentiation and positive regulation of T cell activation, which are concerned with antivirus.

The KEGG category enrichment analysis (see Additional file 6) illustrates that three candidate biomarkers (LCK, lymphocyte-specific protein tyrosine kinase; KLRK1, killer cell lectin-like receptor subfamily K, member 1; TNFSF10, tumor necrosis factor (ligand) superfamily, member 10) are components of the Natural killer cell mediated cytotoxicity pathway, which is important in antineoplastic, antivirus and immune regulation. It has been reported that impairment of natural killer cell activity is associated with chronic hepatitis C virus infection[14].

## Discussion

It is evident that the prediction accuracy of CA patients is higher than AA patients. Constitutively different responses of black and white hepatitis C patients to pegylated interferon and ribavirin therapy had been reported[15] before our study. It shows in our study as, markers that may be important for predicting response did not change as remarkably in AA patients as in CA patients. Our diagnostic model did not perform well on AA patients who did not demonstrate sufficient differentially expressed genes at early time points.

Our results implied that if there is a sensitive change of gene expression profile at early treatment time points, the diagnostic model will be more sensitive and useful. This was confirmed by the simplified diagnostic model. With day 1 model CA patients with HCV infection who are more responsive to interferon and ribavirin therapy can already be predicted of their treatment outcome after 28 days. With less sensitive response the diagnostic model may need to be stretched to include profiles after longer time treatment, like in AA patients. Even with AA patients, profiles of early time points are sufficient for making reasonable predictions of outcome. This is good news for clinical and experimental workers. It means that the strategy of using early time-treatment gene expression profiling to predict outcome for potential long-term treatment is affordable and applicable.

Microarray gene expression analysis has been proved valuable in numerous applications including disease classification, diagnosis, survival analysis, choice of therapy etc, but rarely for more complex clinical problems such as the dynamic prediction of treatment effects we addressed in this paper. We tried several methods including traditional statistical techniques and the latest computer-intensive techniques to predict the final treatment effects based on the static gene expression profiles before treatment and the prediction results were unacceptable. Dynamic prediction chains using time series gene expression profiles have been proved to make more successful prediction model. There were two outcome prediction studies based on 70-gene expression dataset generated by kinetic reverse-transcription PCR from 52 multiple sclerosis patients treated with rIFNβ[16,17]. They obtained good results but were limited to the 70 genes. Our model directly applied on large-scale microarray data, and may have found some novel biomarkers. Our results justify further biological studies to evaluate whether these candidate biomarkers could truly predict the effect of interferon and ribavirian therapy. Further investigations may shed light on the mechanisms of different responses between CA patients and AA patients of HCV infection to this kind of therapy.

## Conclusion

Our time-dependent diagnostic model suggests a way of using time series gene expression profiling to predict the treatment effect of pegylated interferon and ribavirin therapy on HCV infected patients. Similar experimental and bioinformatics strategies may be used to improve treatment decisions for other chronic diseases. This may be an important strategy in future personalized medicine.

## Authors' contributions

TH and KT carried out the study. TH and LX wrote the manuscript. LX and YxL supervised the project. YS provided statistical suggestions, CcW contributed discussions. All authors read and approved the final manuscript.

## Supplementary Material

Additional file 1 Processed microarray data, R code and results of time-dependent diagnostic model (part 1). Additional file 1 and file 2 should be downloaded together. R code is for the performance of model construction. Click here for file

Additional file 2 Processed microarray data, R code and results of time-dependent diagnostic model (part 2). Additional file 1 and file 2 should be downloaded together. R code is for the performance of model construction. Click here for file

Additional file 3 Detailed probe information of thirty candidate biomarkers. The probe information comes from the original microarray probe set. Click here for file

Additional file 4 Dynamic expression graphics of thirty candidate biomarkers. For each of the thirty candidate biomarkers a graph of its expression levels in four groups of patients (good CA, poor CA, good AA, poor AA) at all time points is given. Click here for file

Additional file 5 Literature networks of thirty candidate biomarkers in relation to IFN (Interferon)/HCV (Hepatitis C viruses). The six genes that have direct connections with both IFN and HCV are framed with blue boxes. Click here for file

Additional file 6 GO and KEGG category enrichment analyses of thirty candidate biomarkers. Thirty-seven enriched GO biological processes and ten GO molecular functions as well as one enriched KEGG pathway are shown (p < 0.01). Click here for file
