# Supplementary material for: The prediction of interferon treatment effects based on time series microarray gene expression profiles
Source: J Transl Med. 2008 Aug 9;6:44. doi: 10.1186/1479-5876-6-44 (PMC2546378; doi:10.1186/1479-5876-6-44)
Supplement: Additional file 4 — Dynamic expression graphics of thirty candidate biomarkers. For each of the thirty candidate biomarkers a graph of its expression levels in four groups of patients (good CA, poor CA, good AA, poor AA) at all time points is given. [file 1479-5876-6-44-S4.pdf]

# 200023_s_at EIF3F
# eukaryotic translation initiation factor 3, subunit F

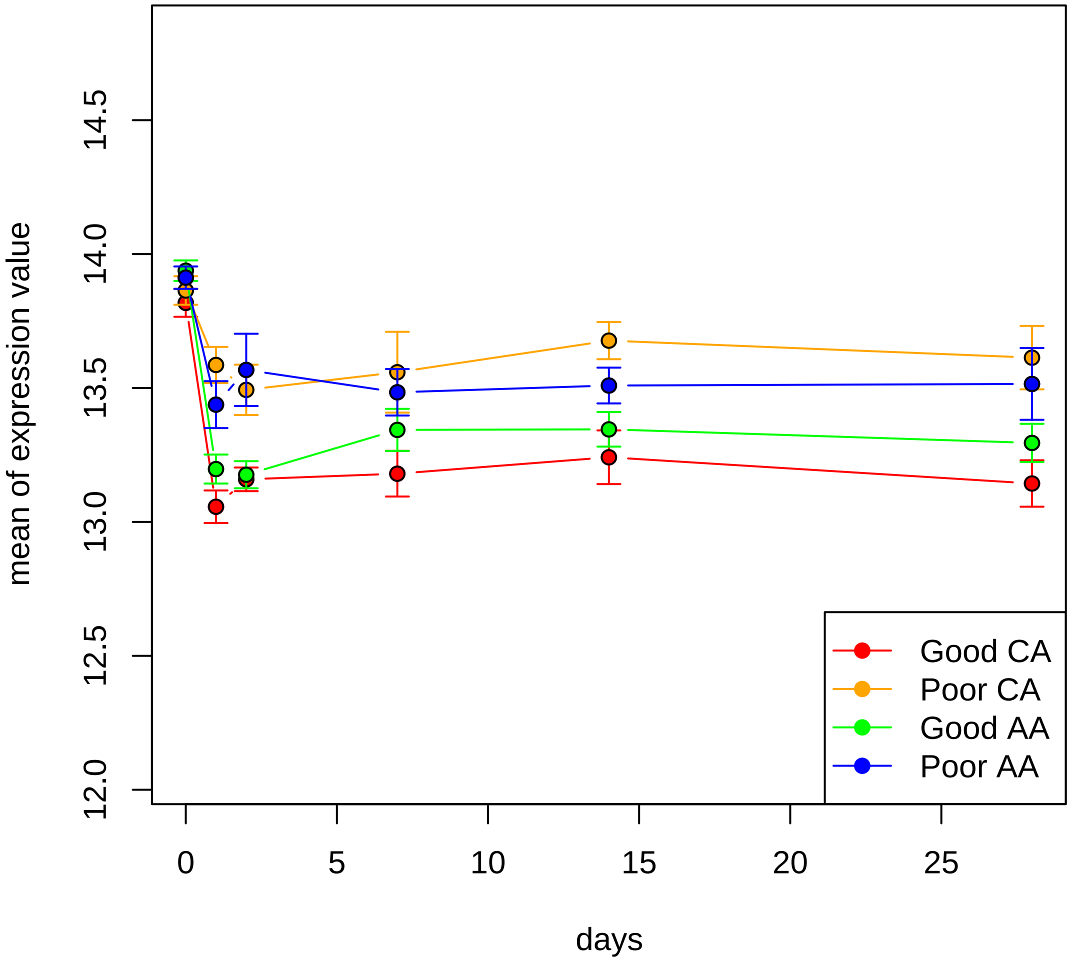

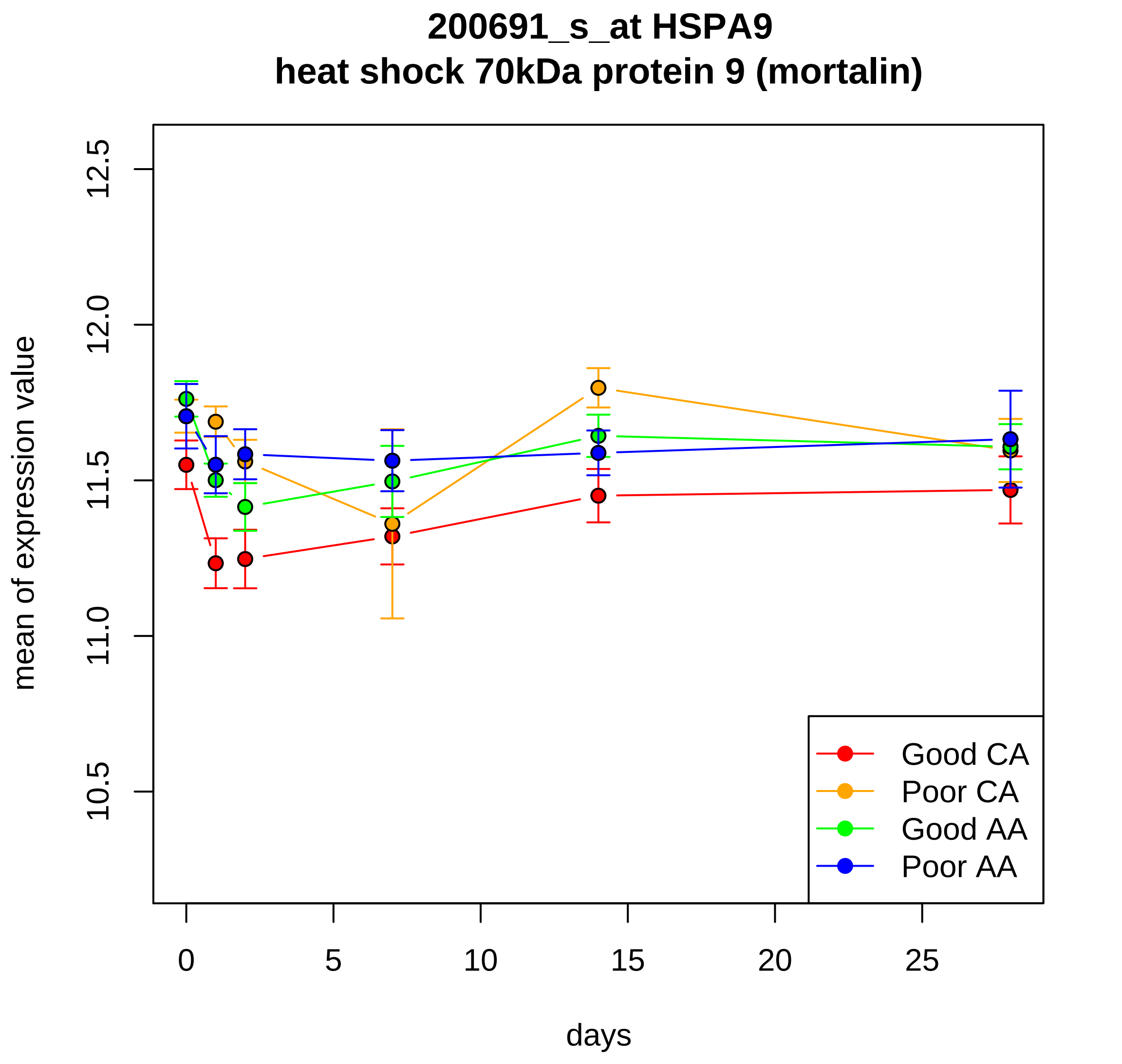
200691_s_at HSPA9
heat shock 70kDa protein 9 (mortalin)
mean of expression value
12.5
12.0
11.5
11.0
10.5
0
5
10
15
20
25
days
Good CA
Poor CA
Good AA
Poor AA

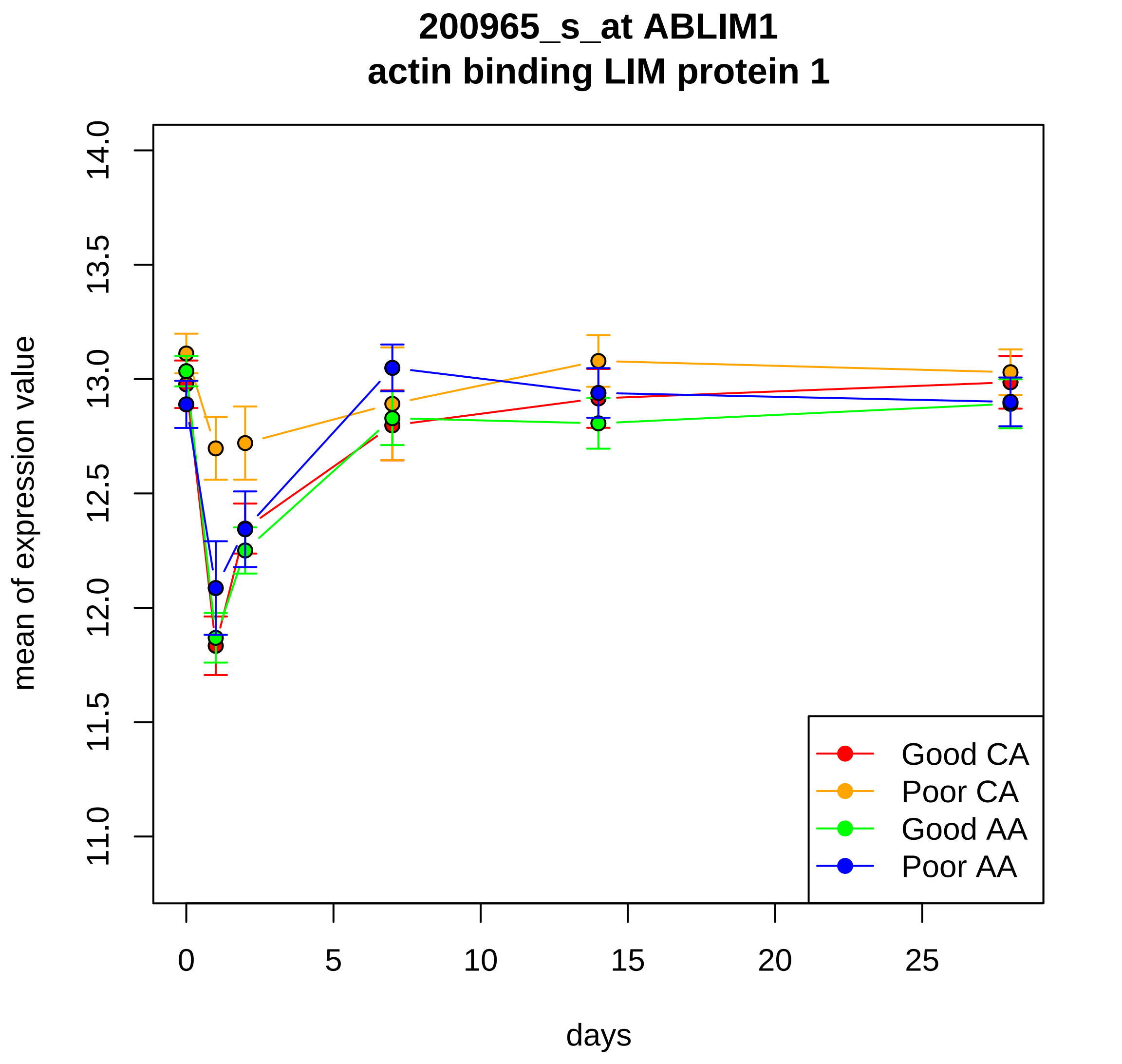

200965_s_at ABLIM1
actin binding LIM protein 1
mean of expression value
days
11.0
11.5
12.0
12.5
13.0
13.5
14.0
0
5
10
15
20
25
Good CA
Poor CA
Good AA
Poor AA

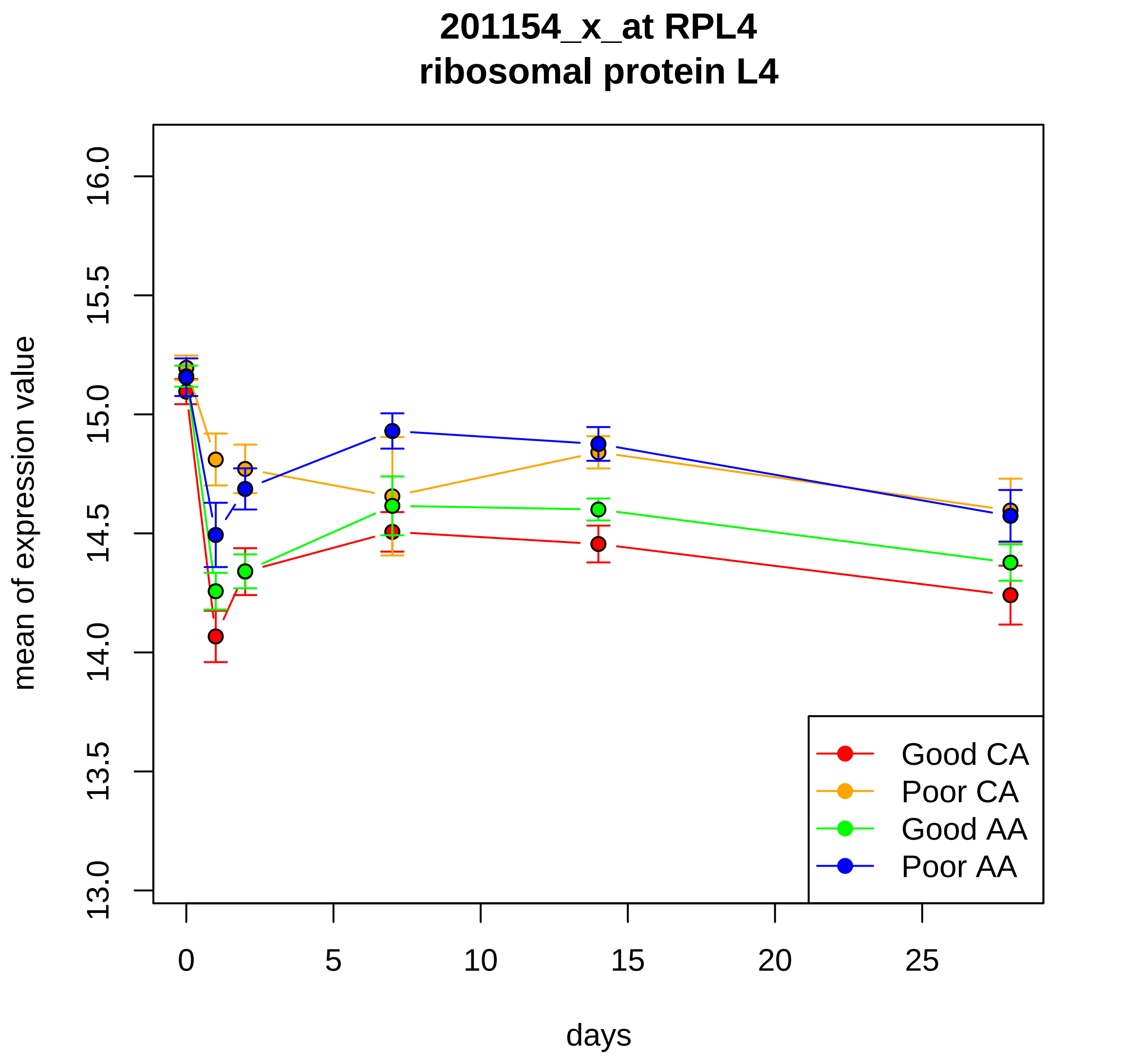

201154_x_at RPL4
ribosomal protein L4
mean of expression value
16.0
15.5
15.0
14.5
14.0
13.5
13.0
0
5
10
15
20
25
days
Good CA
Poor CA
Good AA
Poor AA

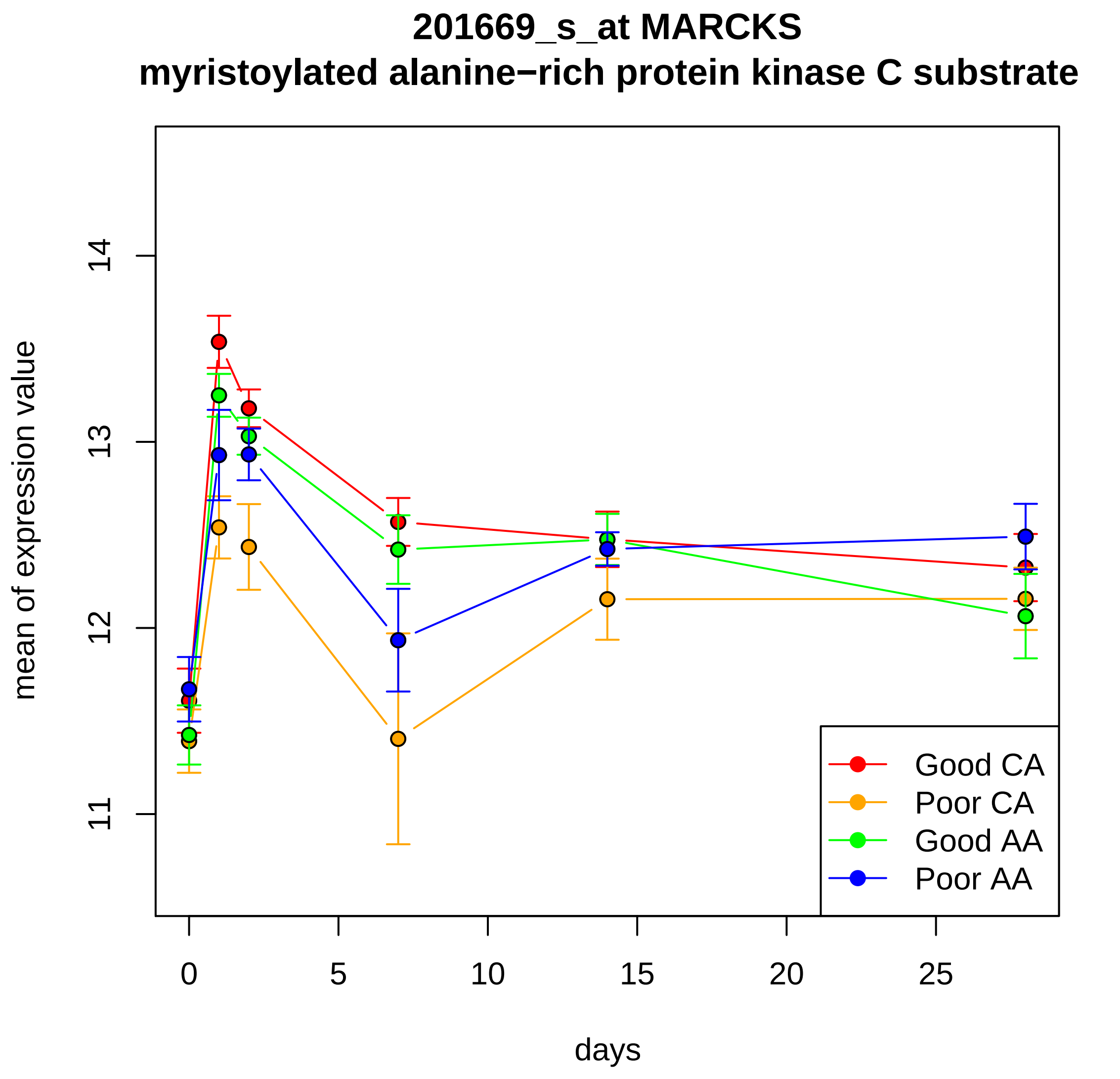

201669_s_at MARCKS
myristoylated alanine−rich protein kinase C substrate
mean of expression value
11
12
13
14
days
0
5
10
15
20
25
Good CA
Poor CA
Good AA
Poor AA

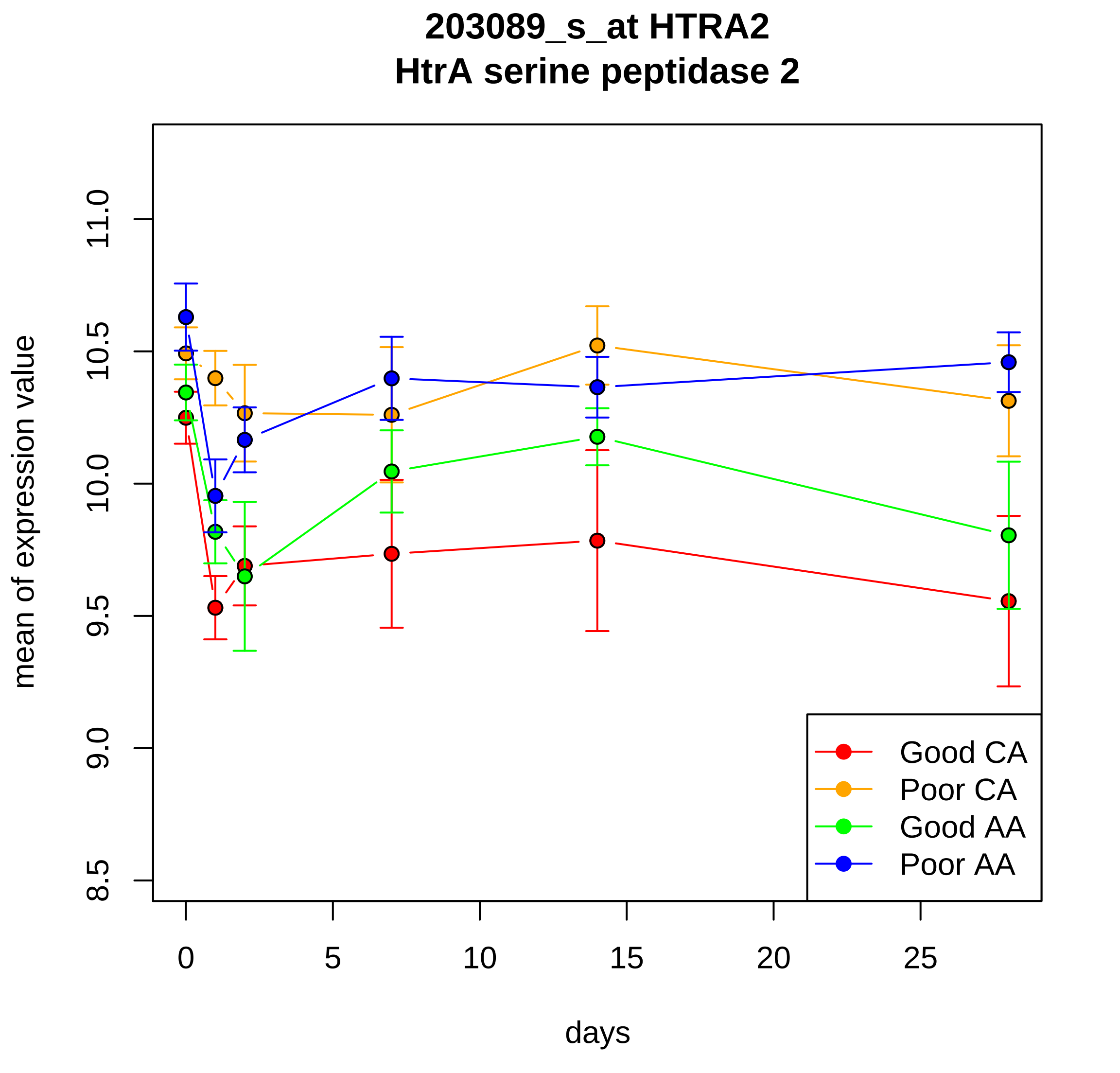
203089_s_at HTRA2
HtrA serine peptidase 2
mean of expression value
8.5
9.0
9.5
10.0
10.5
11.0
0
5
10
15
20
25
days
Good CA
Poor CA
Good AA
Poor AA

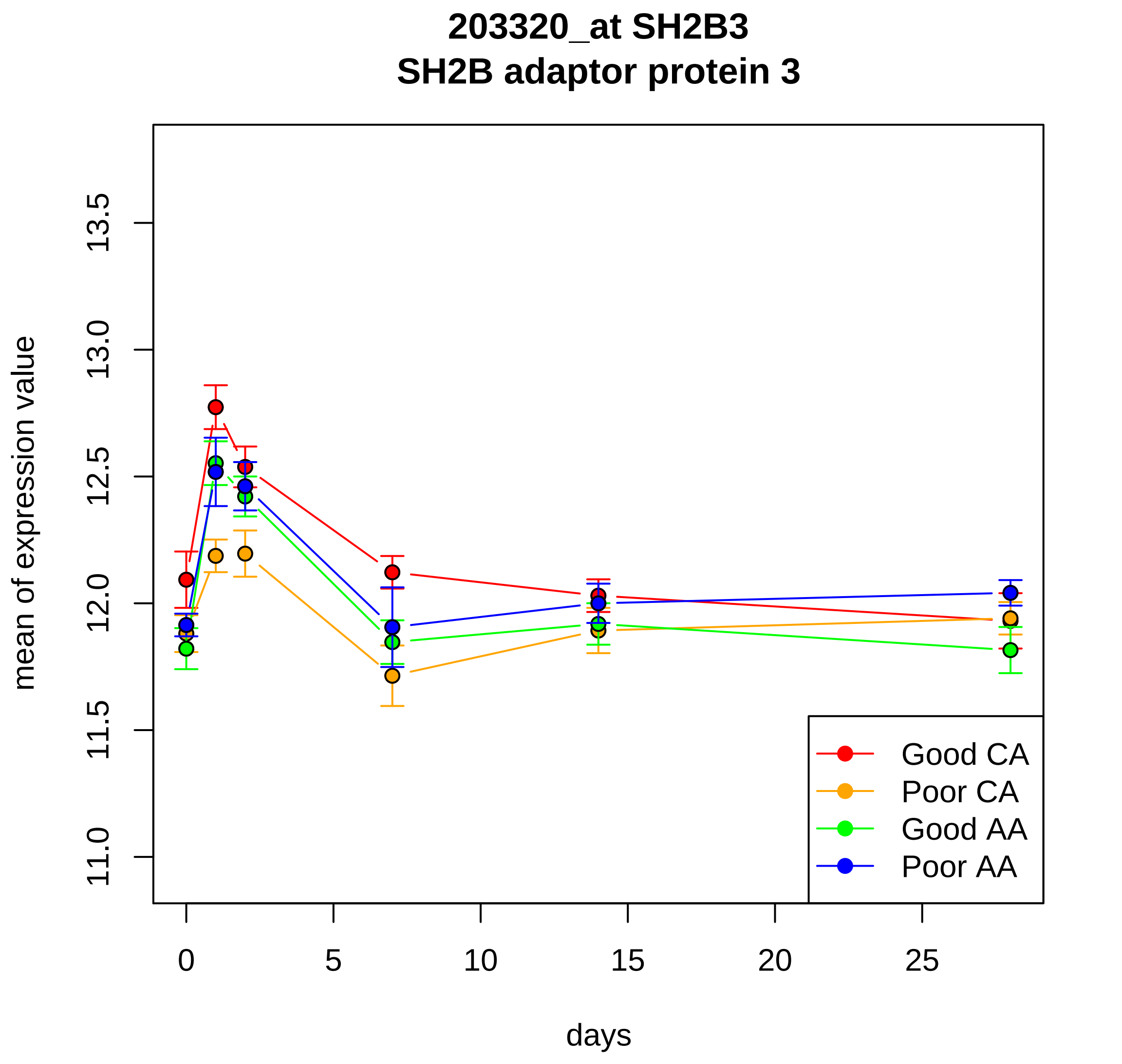

203320_at SH2B3
SH2B adaptor protein 3
mean of expression value
11.0
11.5
12.0
12.5
13.0
13.5
0
5
10
15
20
25
days
Good CA
Poor CA
Good AA
Poor AA

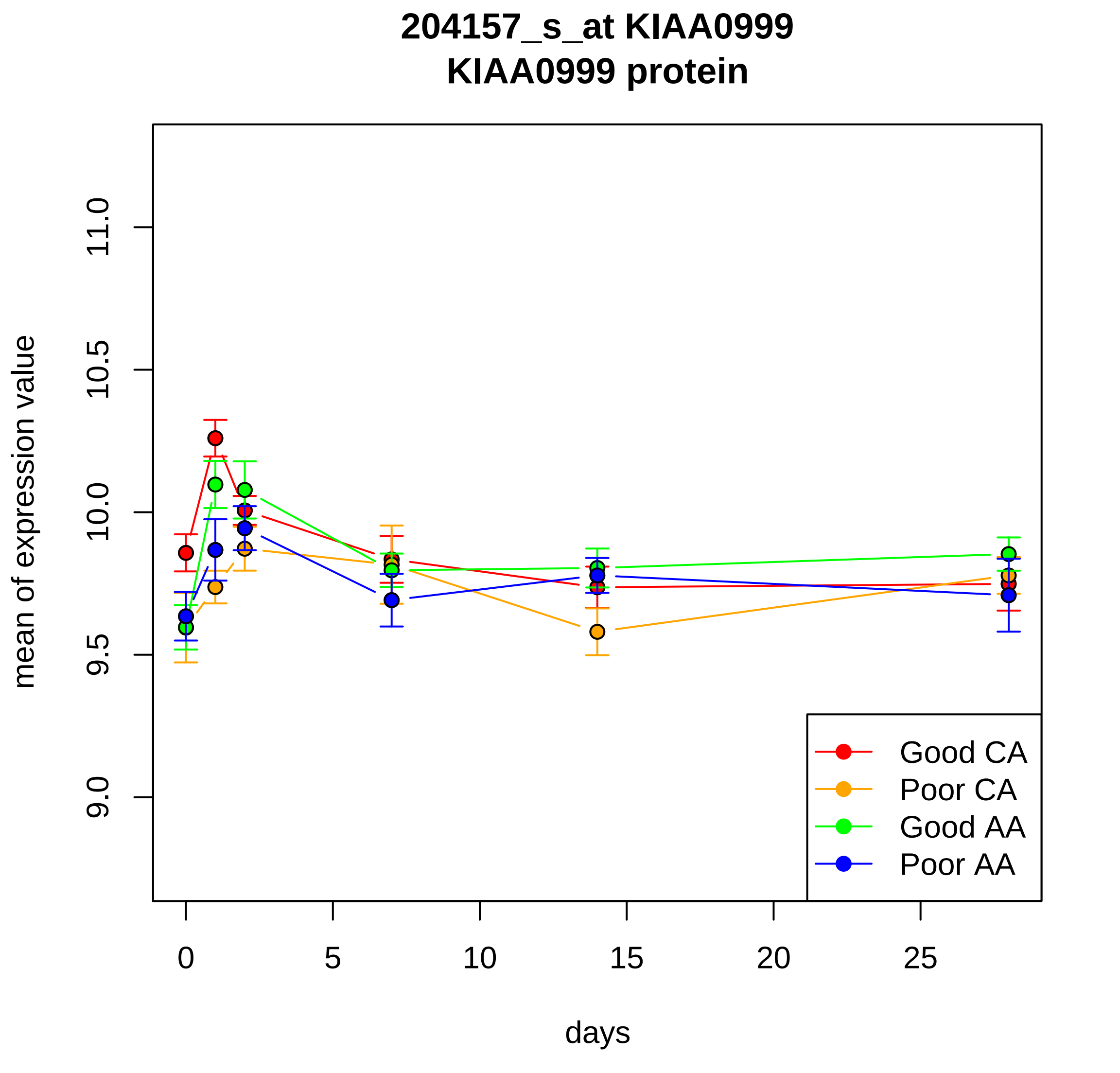

204157_s_at KIAA0999
KIAA0999 protein
mean of expression value
9.0
9.5
10.0
10.5
11.0
0
5
10
15
20
25
days
Good CA
Poor CA
Good AA
Poor AA

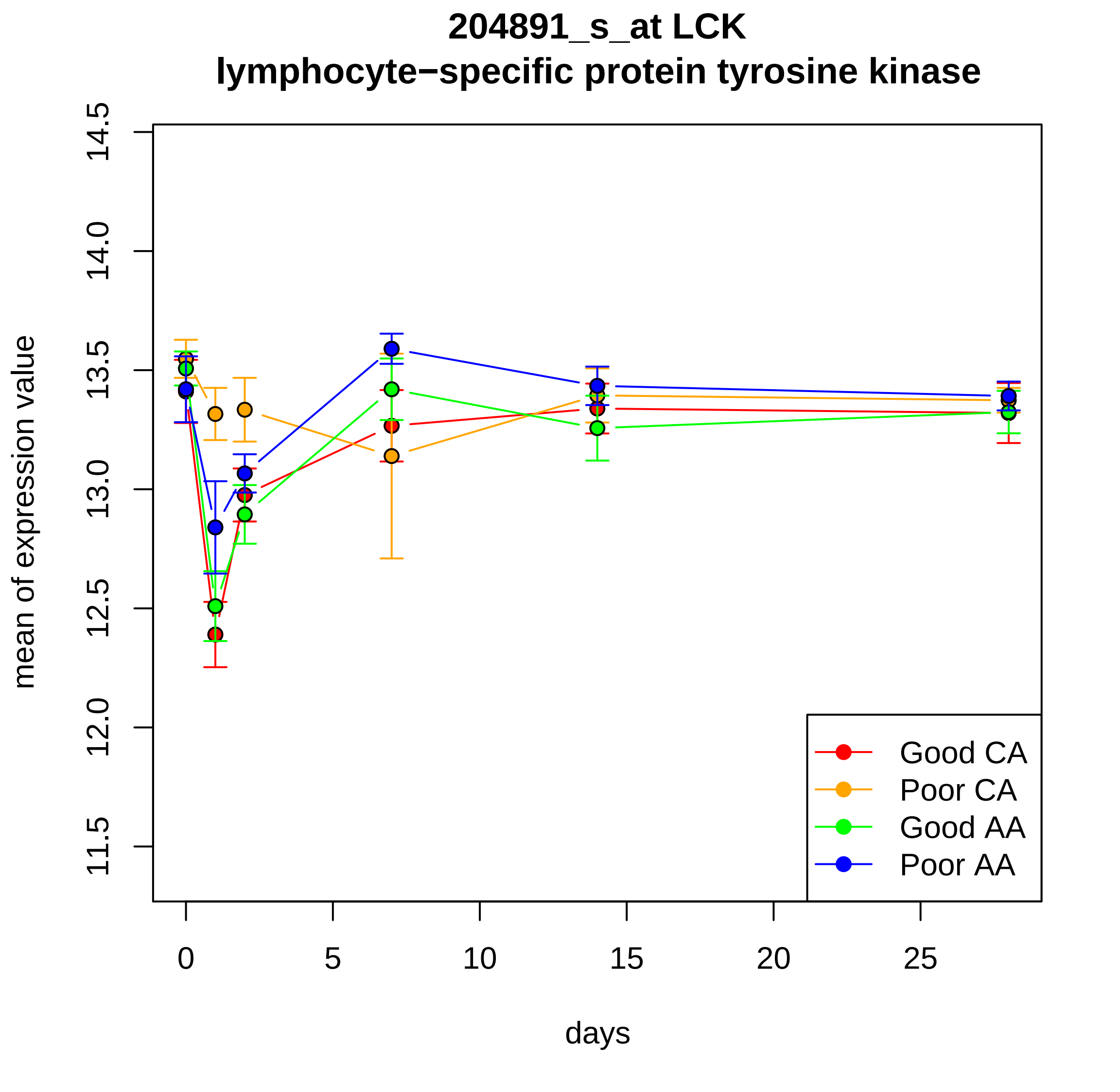

204891_s_at LCK
lymphocyte−specific protein tyrosine kinase
mean of expression value
days
11.5
12.0
12.5
13.0
13.5
14.0
14.5
0
5
10
15
20
25
Good CA
Poor CA
Good AA
Poor AA

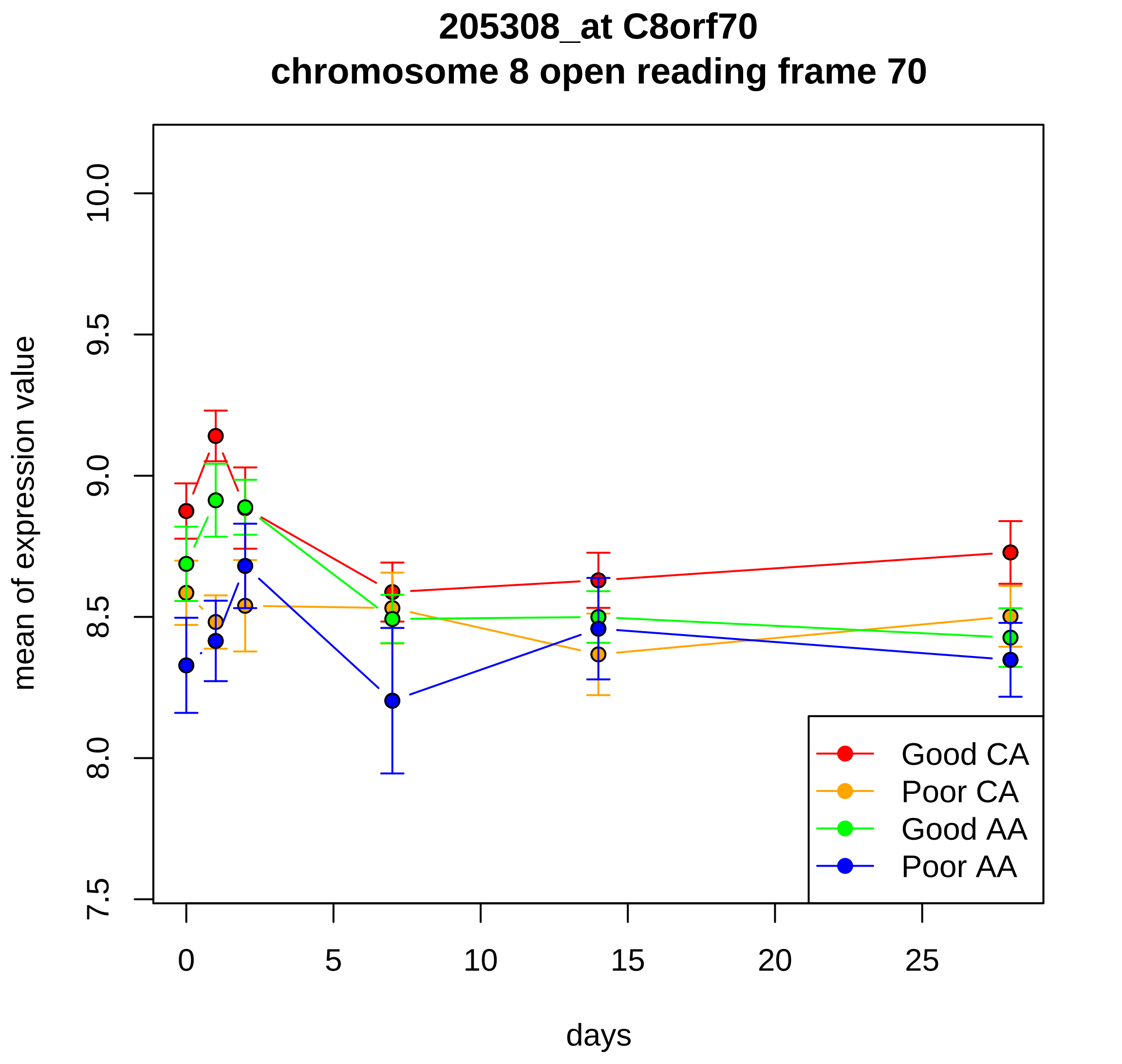

205308_at C8orf70
chromosome 8 open reading frame 70
mean of expression value
7.5
8.0
8.5
9.0
9.5
10.0
0
5
10
15
20
25
days
Good CA
Poor CA
Good AA
Poor AA

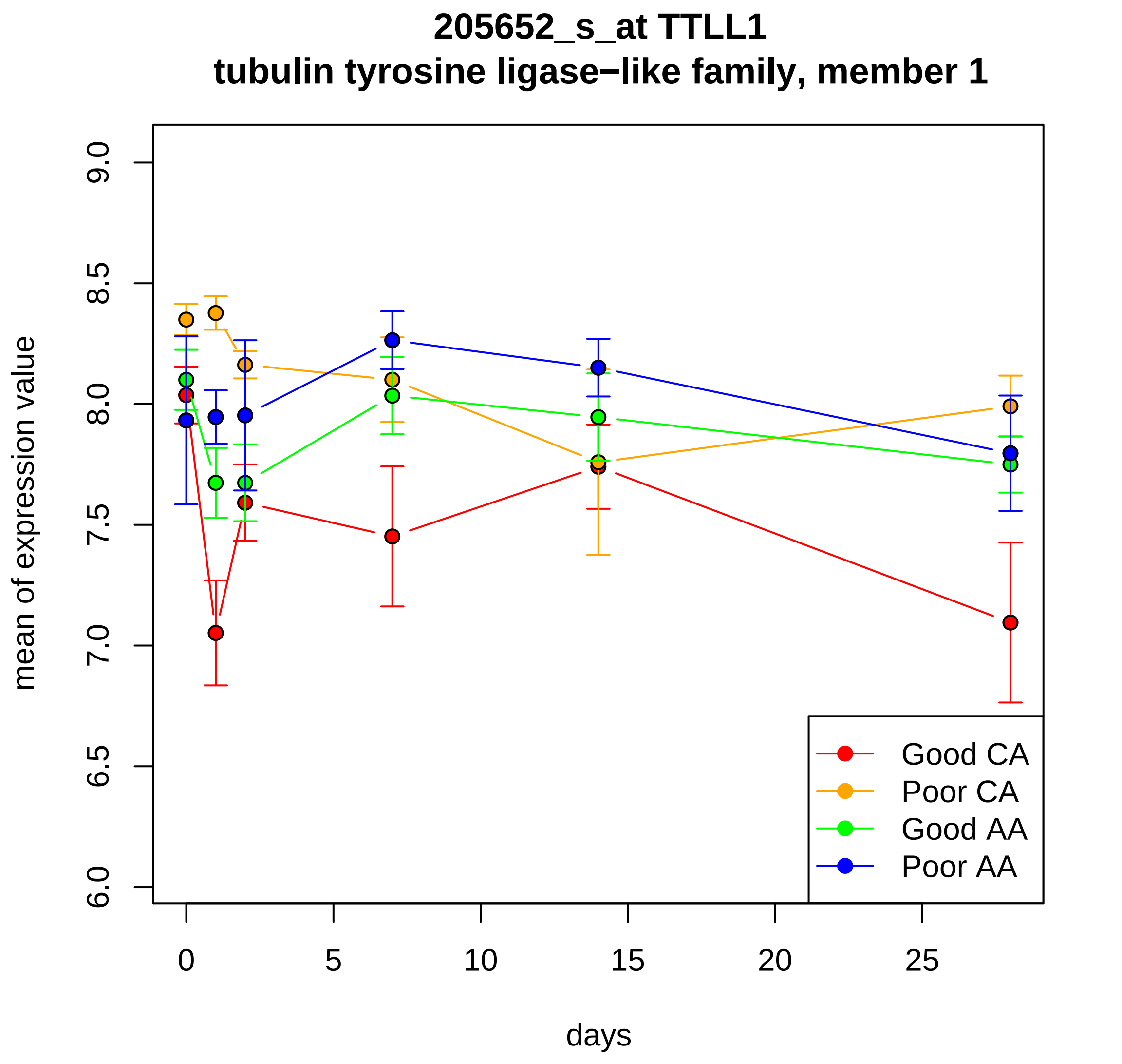

205652_s_at TTLL1
tubulin tyrosine ligase−like family, member 1
mean of expression value
days
0
5
10
15
20
25
6.0
6.5
7.0
7.5
8.0
8.5
9.0
Good CA
Poor CA
Good AA
Poor AA

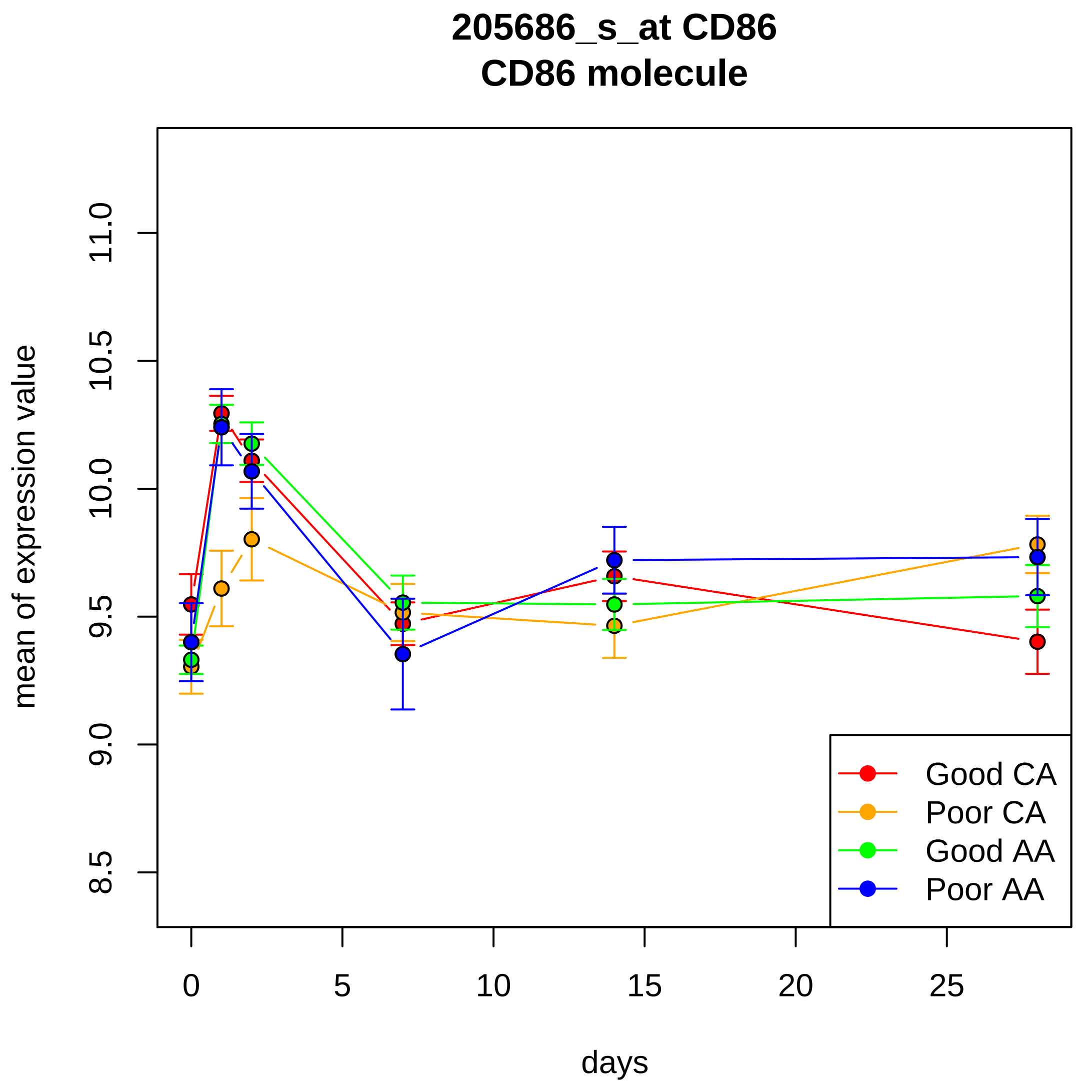

205686_s_at CD86
CD86 molecule
mean of expression value
8.5
9.0
9.5
10.0
10.5
11.0
0
5
10
15
20
25
days
Good CA
Poor CA
Good AA
Poor AA

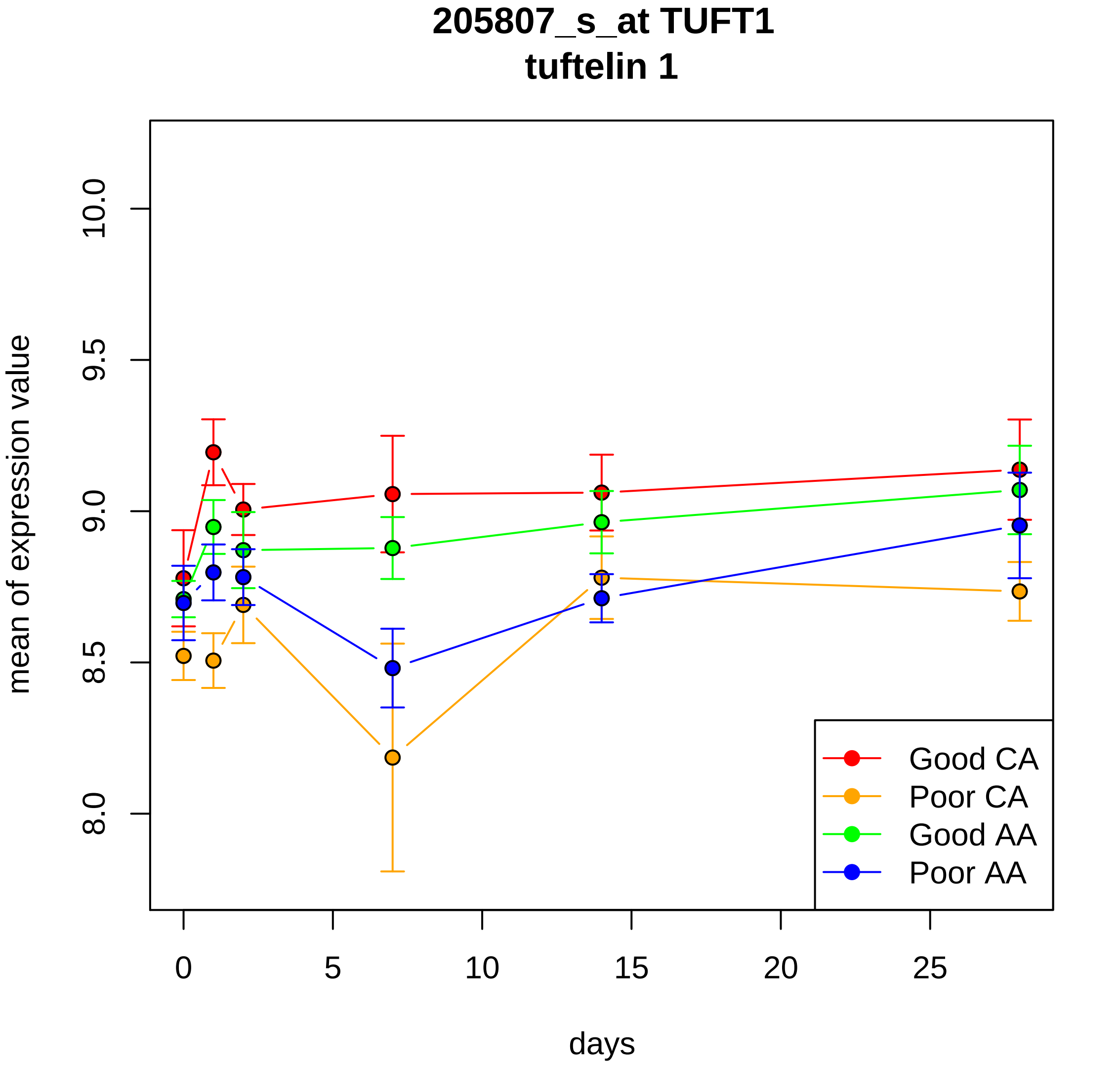
205807_s_at TUFT1
tuftelin 1
mean of expression value
8.0
8.5
9.0
9.5
10.0
0
5
10
15
20
25
days
Good CA
Poor CA
Good AA
Poor AA

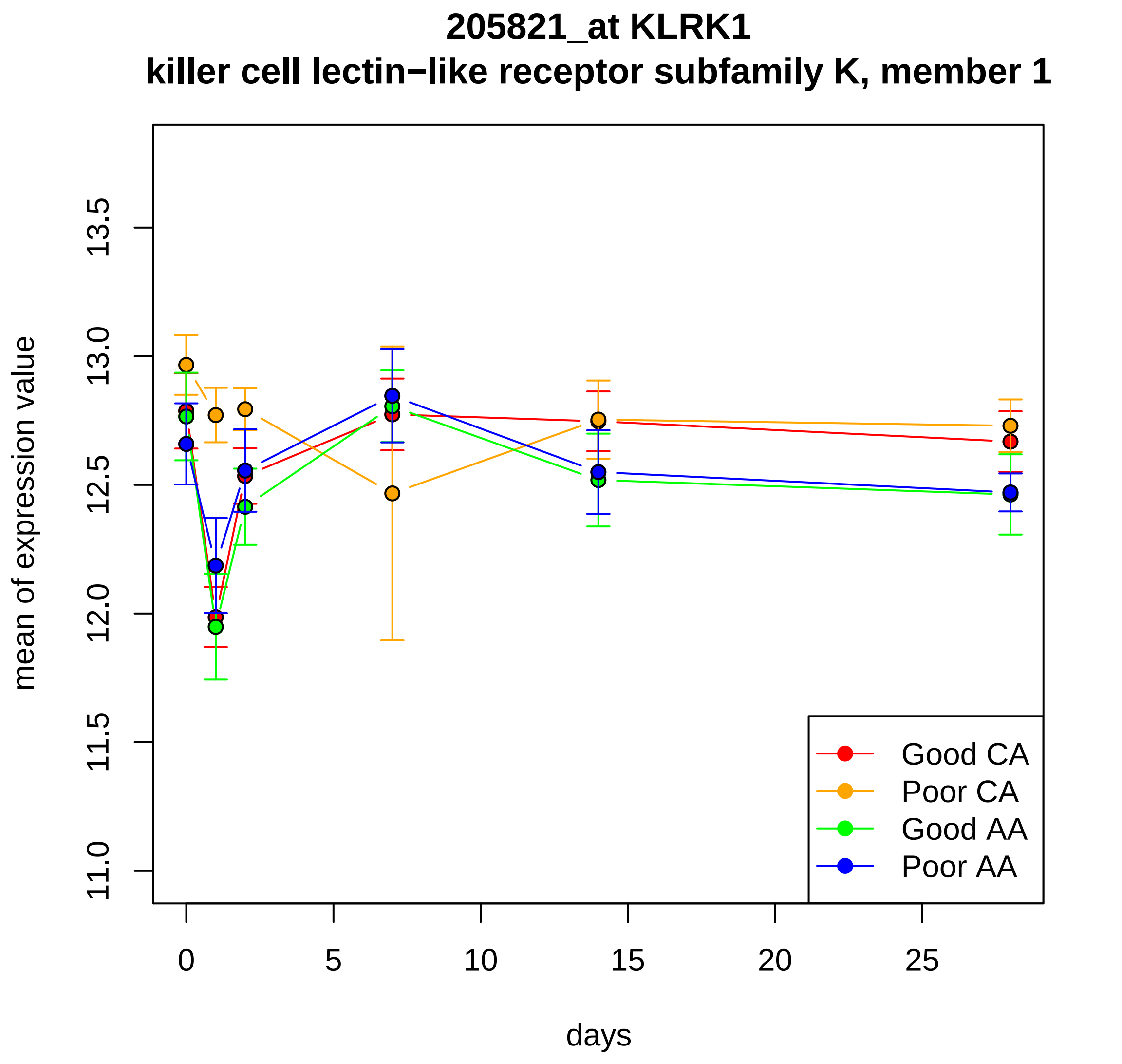

205821_at KLRK1
killer cell lectin−like receptor subfamily K, member 1
mean of expression value
11.0
11.5
12.0
12.5
13.0
13.5
0
5
10
15
20
25
days
Good CA
Poor CA
Good AA
Poor AA

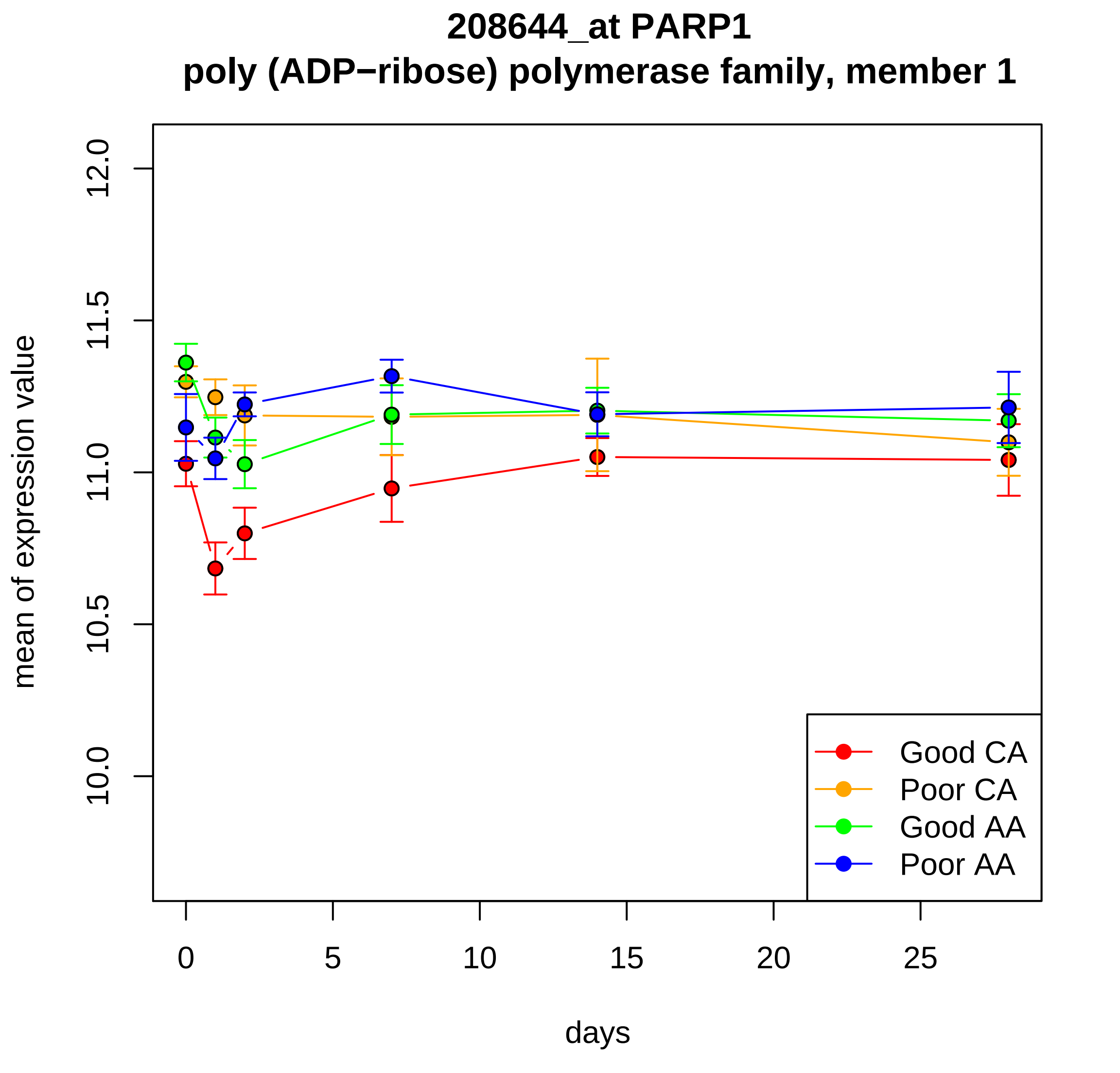

208644_at PARP1
poly (ADP−ribose) polymerase family, member 1
mean of expression value
12.0
11.5
11.0
10.5
10.0
0
5
10
15
20
25
days
Good CA
Poor CA
Good AA
Poor AA

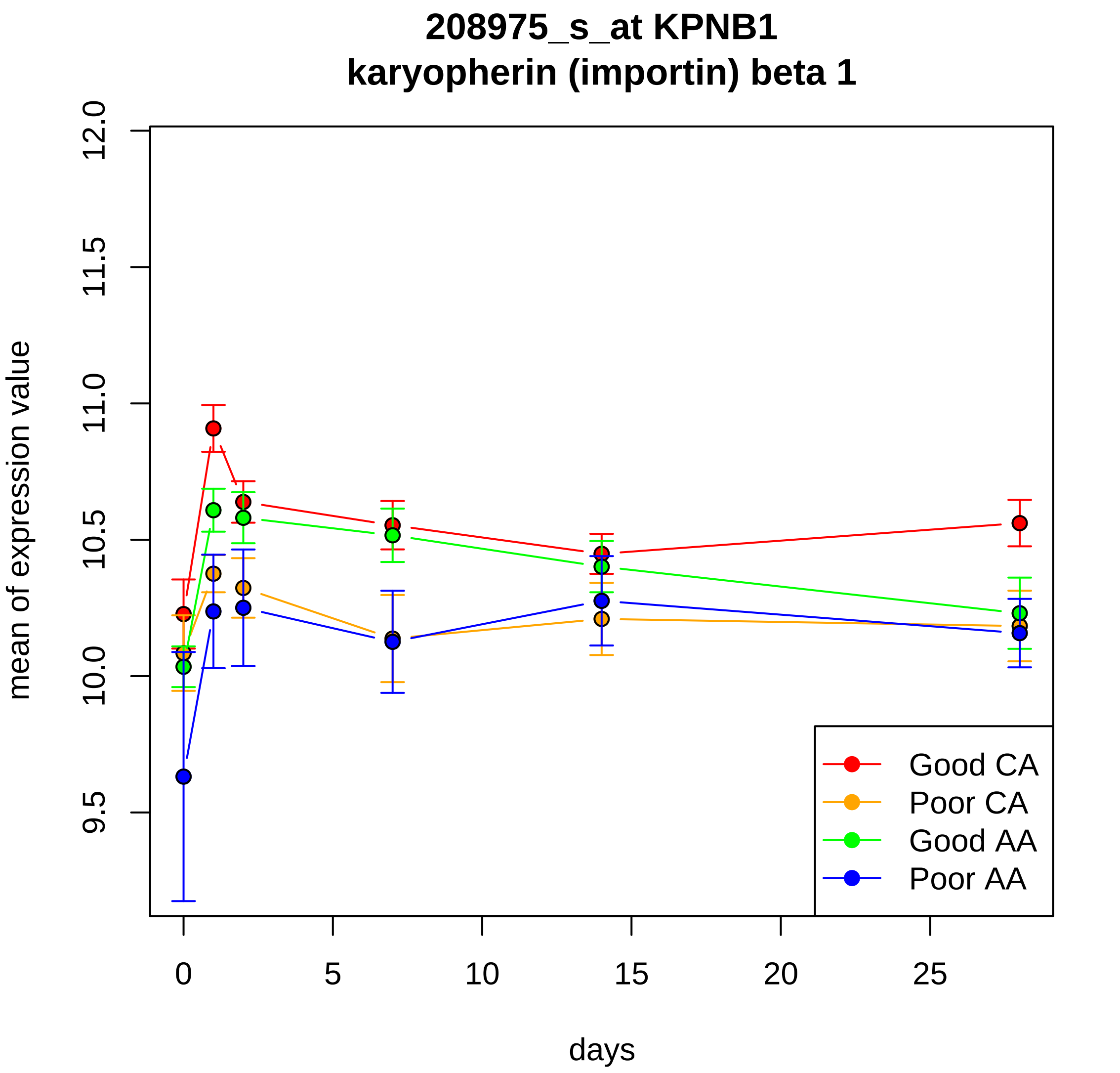

208975_s_at KPNB1
karyopherin (importin) beta 1
mean of expression value
12.0
11.5
11.0
10.5
10.0
9.5
0
5
10
15
20
25
days
Good CA
Poor CA
Good AA
Poor AA

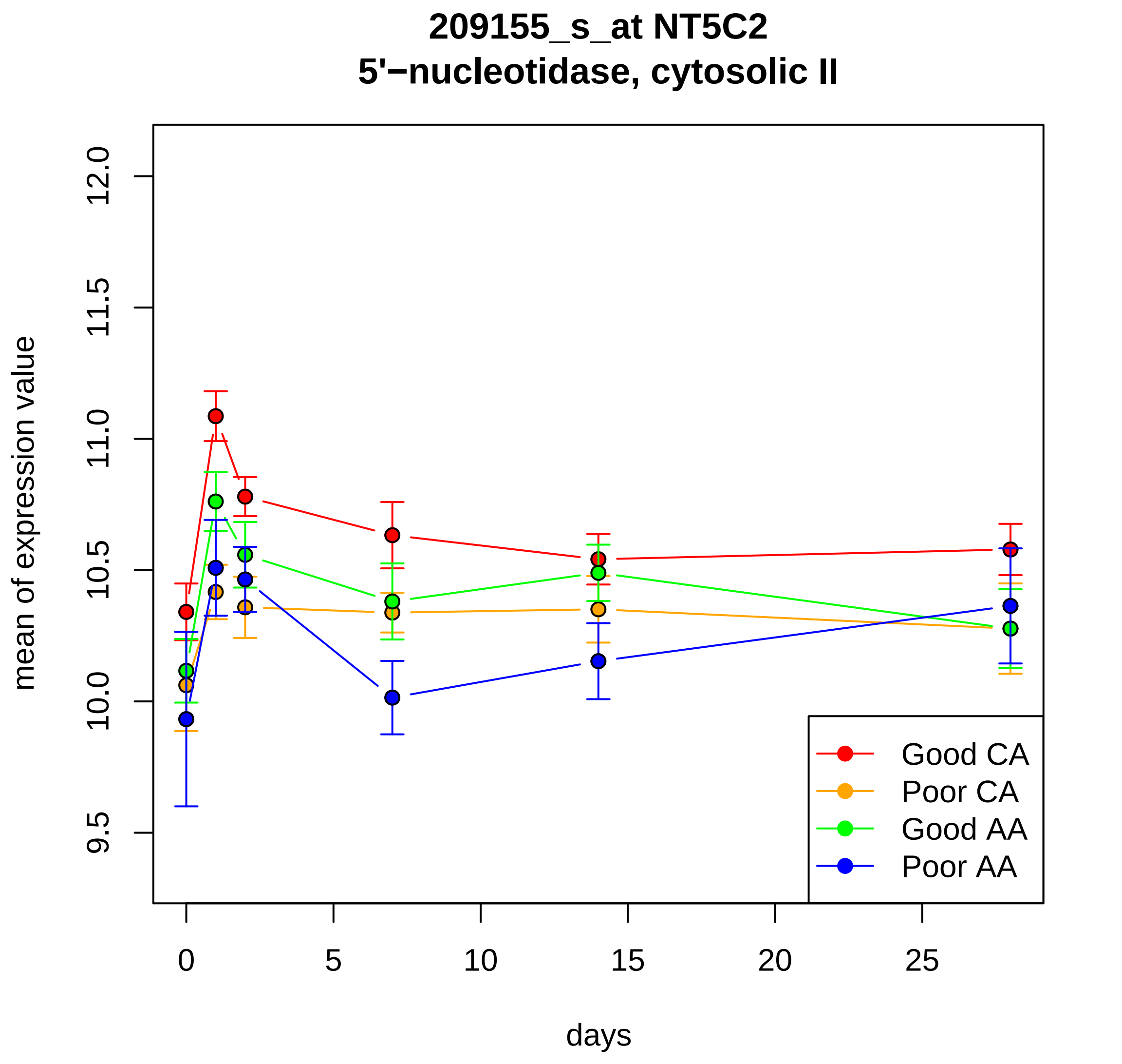

209155_s_at NT5C2
5'−nucleotidase, cytosolic II
mean of expression value
9.5
10.0
10.5
11.0
11.5
12.0
0
5
10
15
20
25
days
Good CA
Poor CA
Good AA
Poor AA

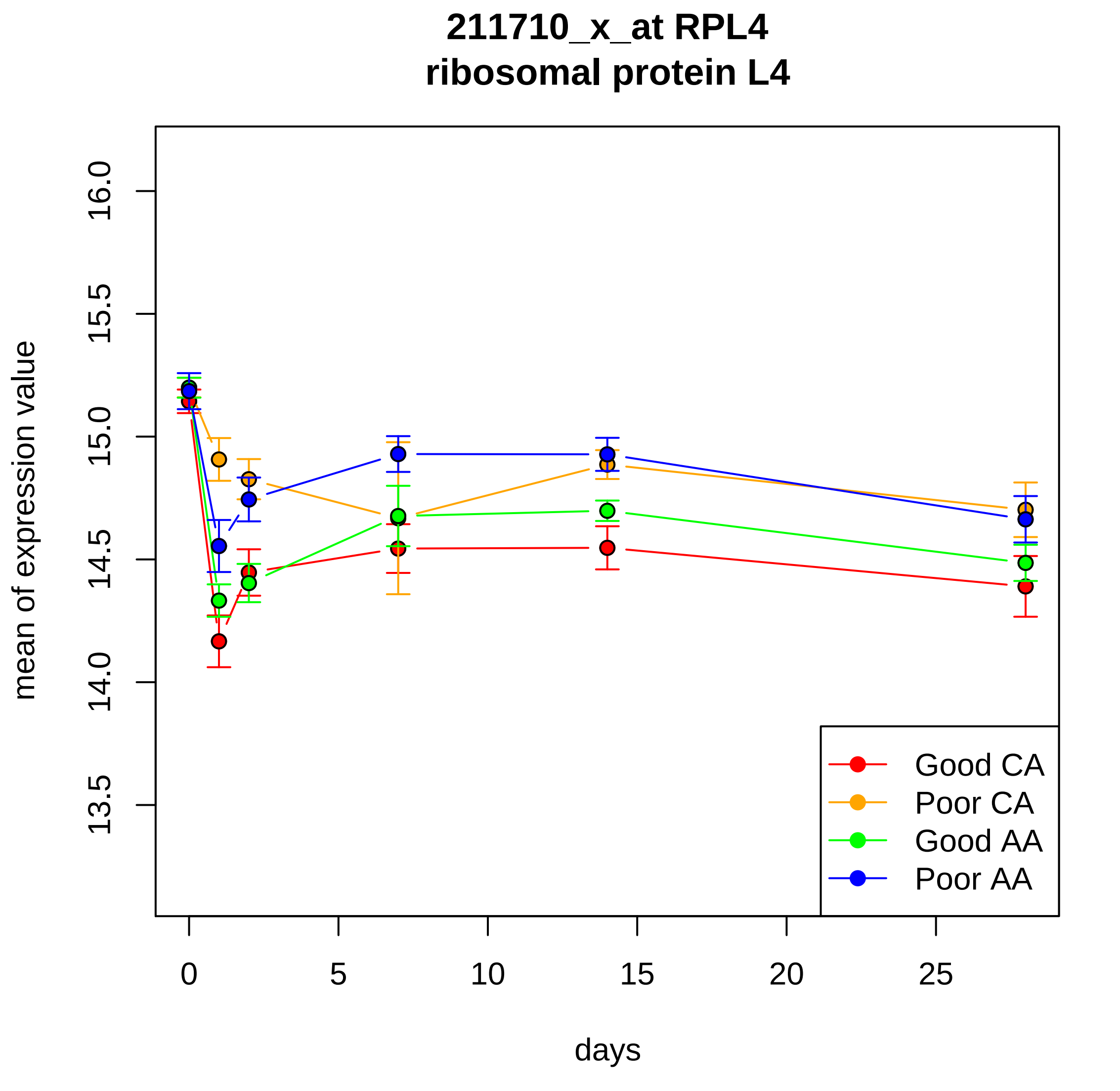

211710_x_at RPL4
ribosomal protein L4
mean of expression value
16.0
15.5
15.0
14.5
14.0
13.5
0
5
10
15
20
25
days
Good CA
Poor CA
Good AA
Poor AA

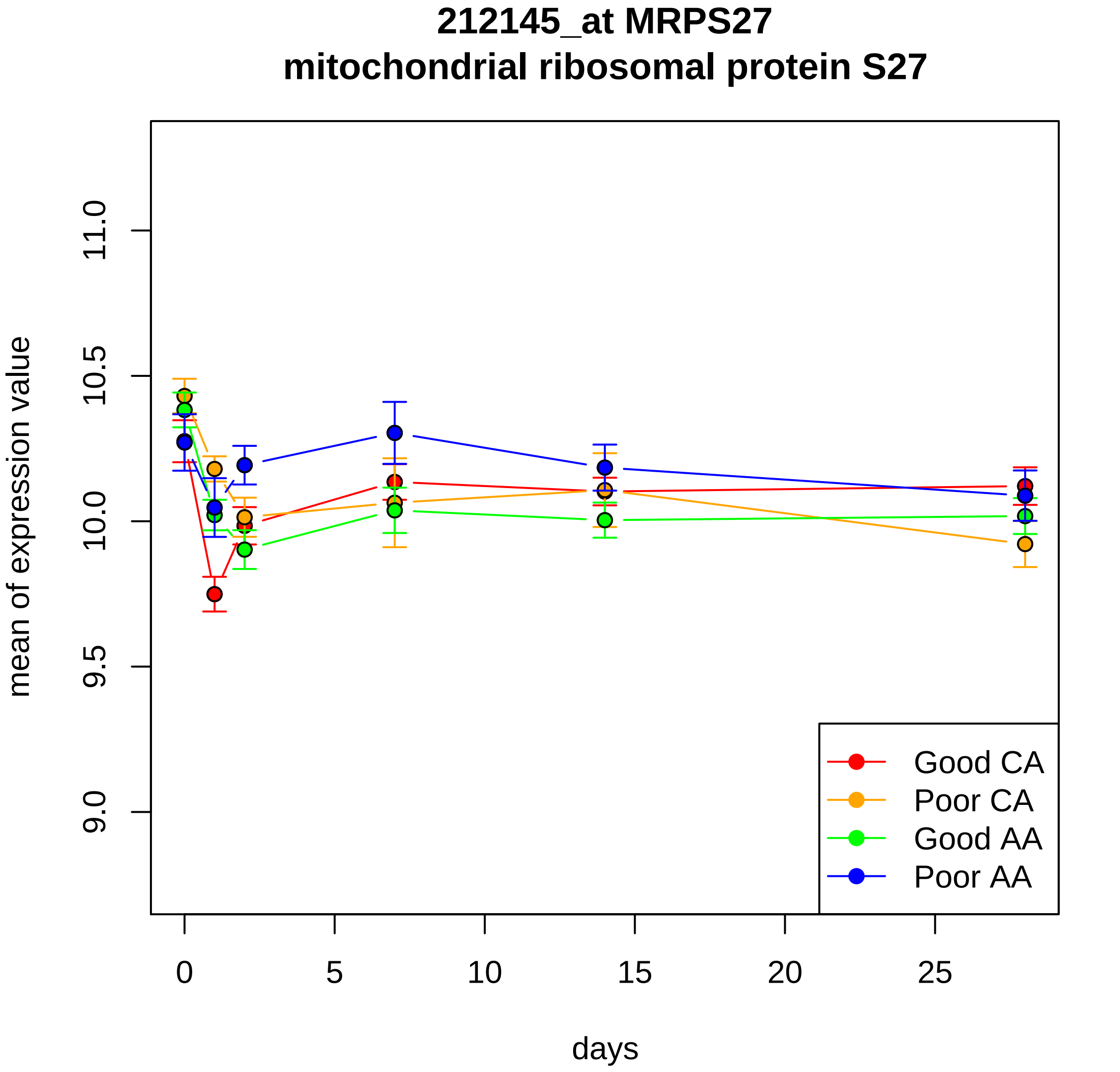

212145_at MRPS27
mitochondrial ribosomal protein S27
mean of expression value
11.0
10.5
10.0
9.5
9.0
0
5
10
15
20
25
days
Good CA
Poor CA
Good AA
Poor AA

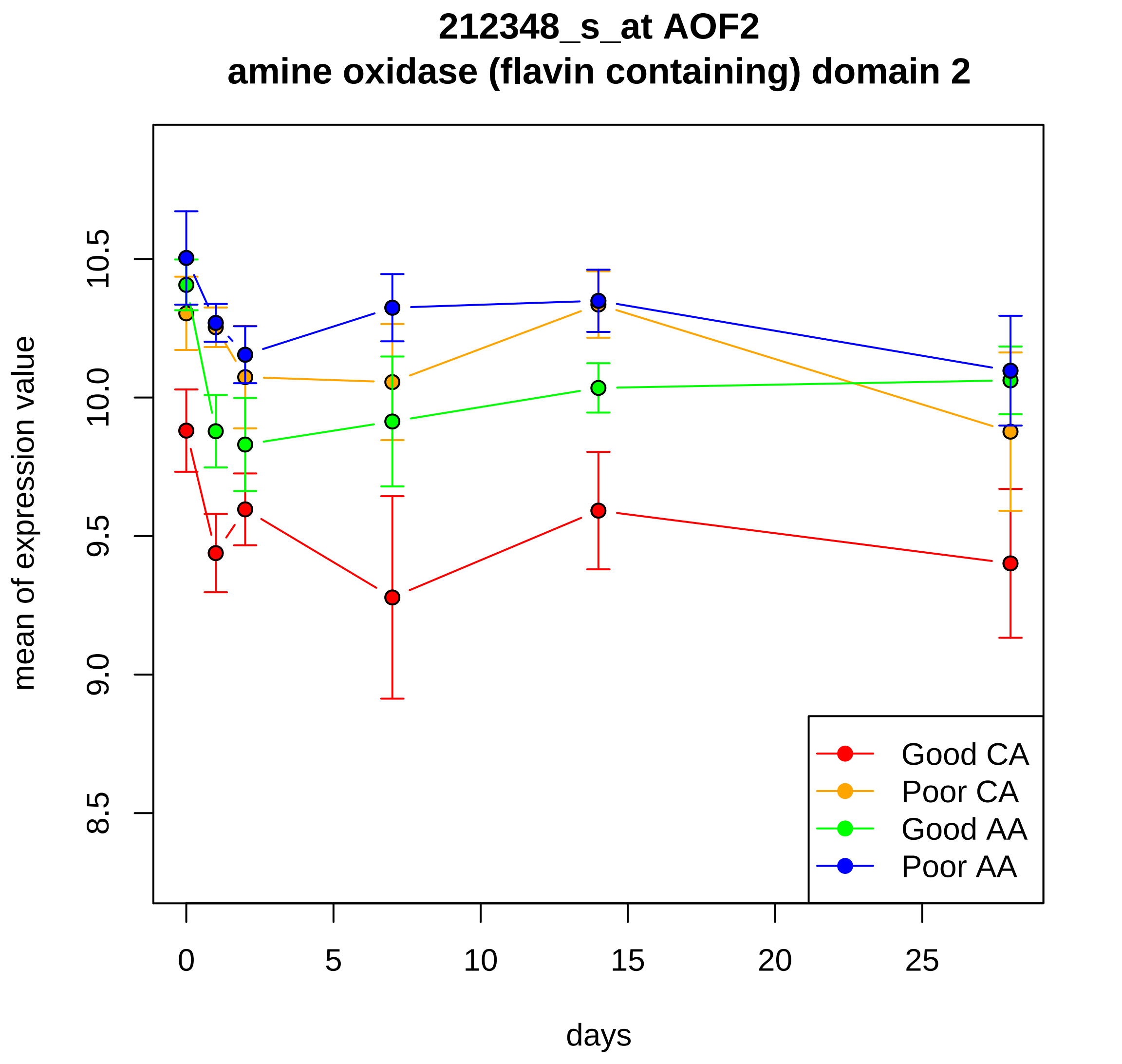
212348_s_at AOF2
amine oxidase (flavin containing) domain 2
mean of expression value
days
10.5
10.0
9.5
9.0
8.5
0
5
10
15
20
25
Good CA
Poor CA
Good AA
Poor AA

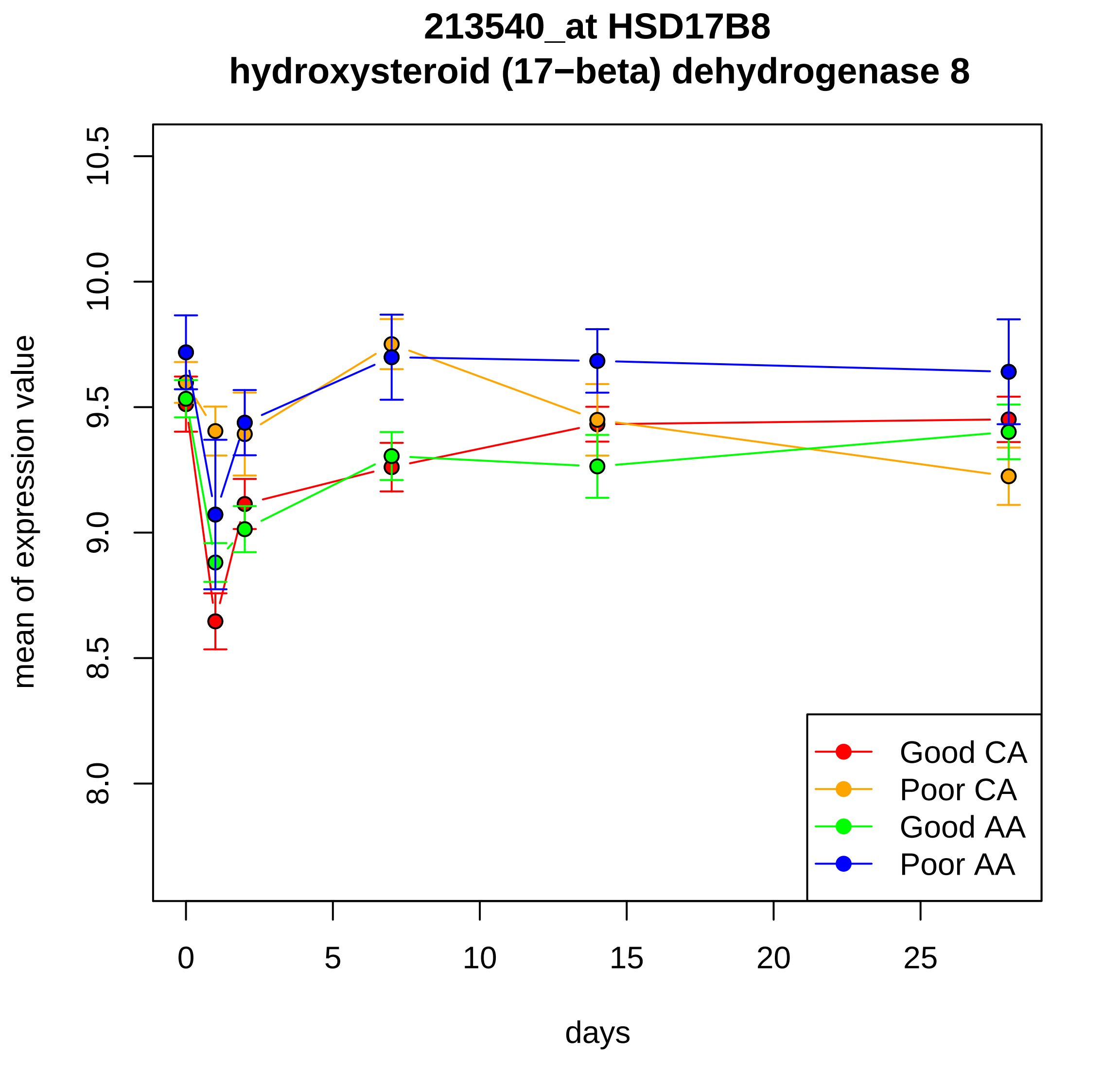

213540_at HSD17B8
hydroxysteroid (17−beta) dehydrogenase 8
mean of expression value
days
8.0
8.5
9.0
9.5
10.0
10.5
0
5
10
15
20
25
Good CA
Poor CA
Good AA
Poor AA

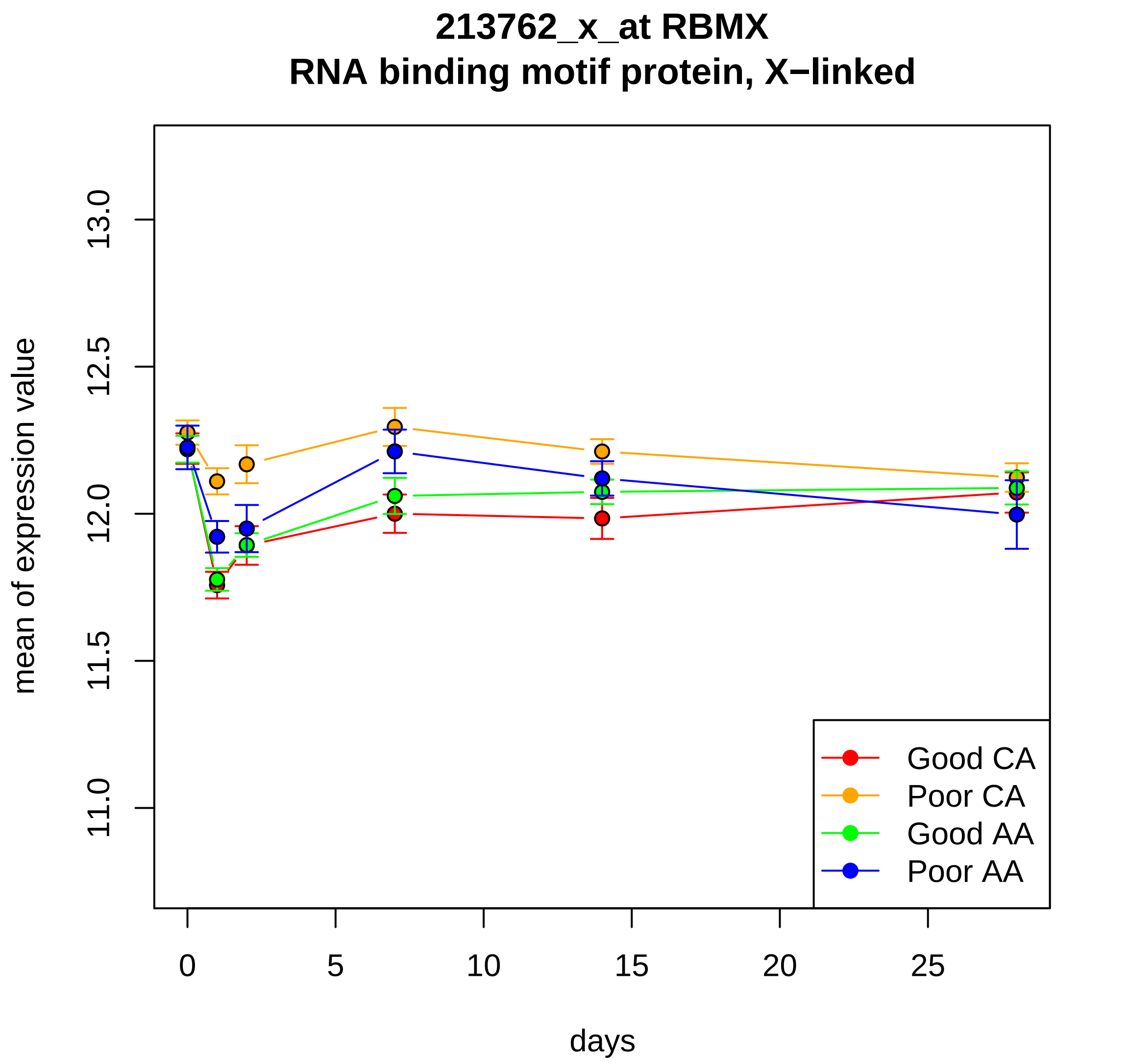

213762_x_at RBMX
RNA binding motif protein, X−linked
mean of expression value
11.0
11.5
12.0
12.5
13.0
0
5
10
15
20
25
days
Good CA
Poor CA
Good AA
Poor AA

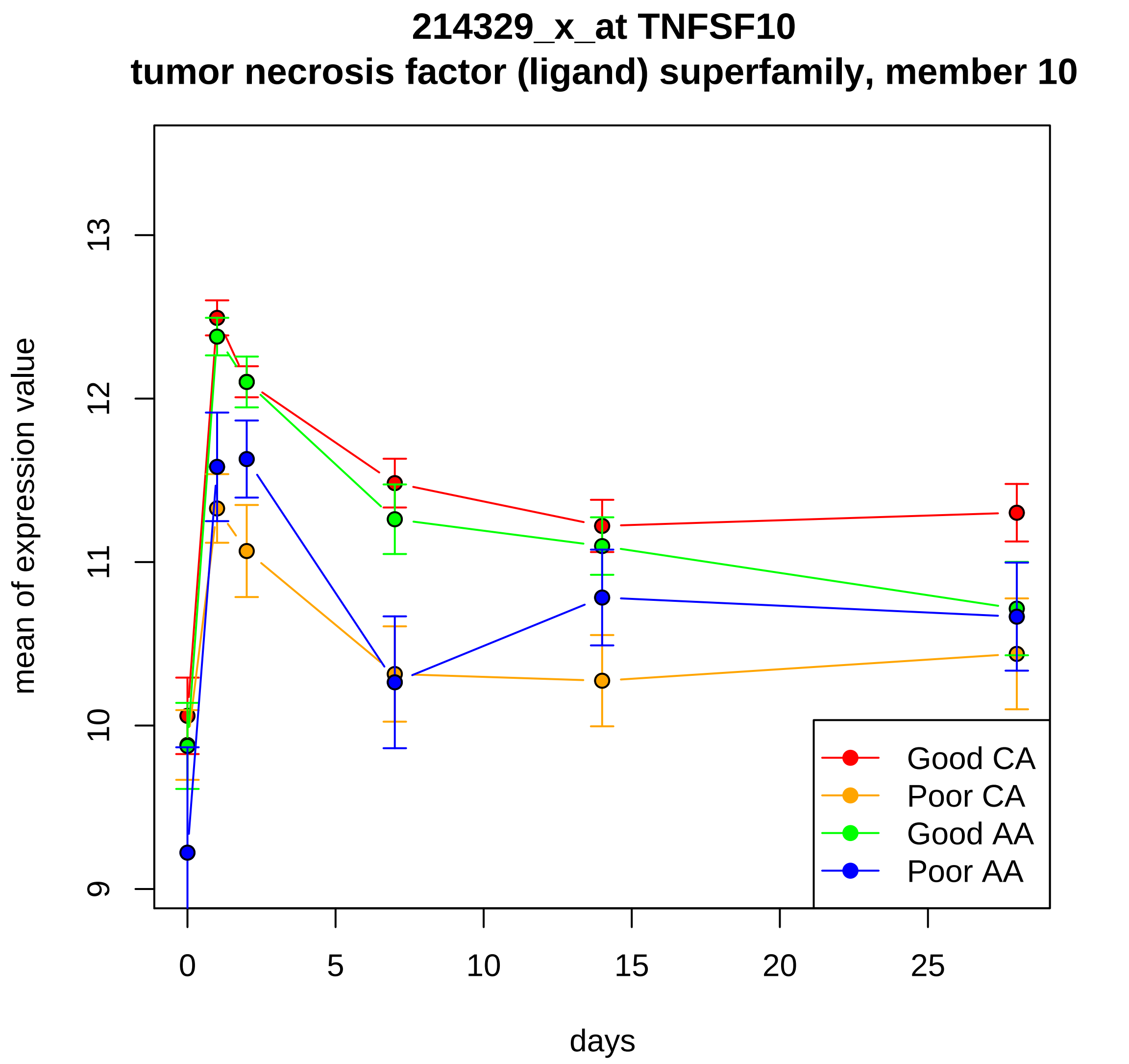

214329_x_at TNFSF10
tumor necrosis factor (ligand) superfamily, member 10
mean of expression value
9
10
11
12
13
0
5
10
15
20
25
days
Good CA
Poor CA
Good AA
Poor AA

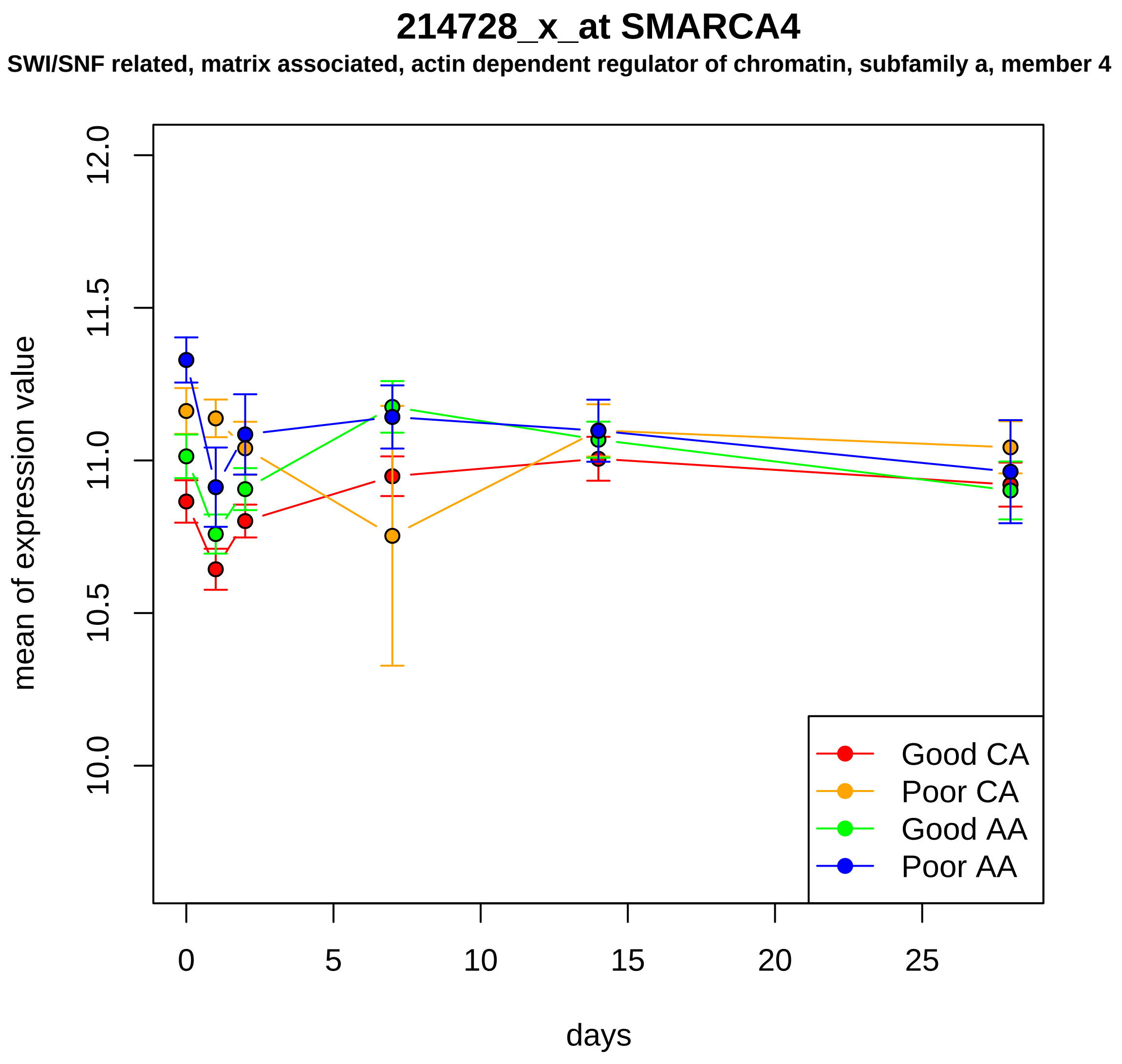

214728_x_at SMARCA4
SWI/SNF related, matrix associated, actin dependent regulator of chromatin, subfamily a, member 4
mean of expression value
10.0
10.5
11.0
11.5
12.0
0
5
10
15
20
25
days
Good CA
Poor CA
Good AA
Poor AA

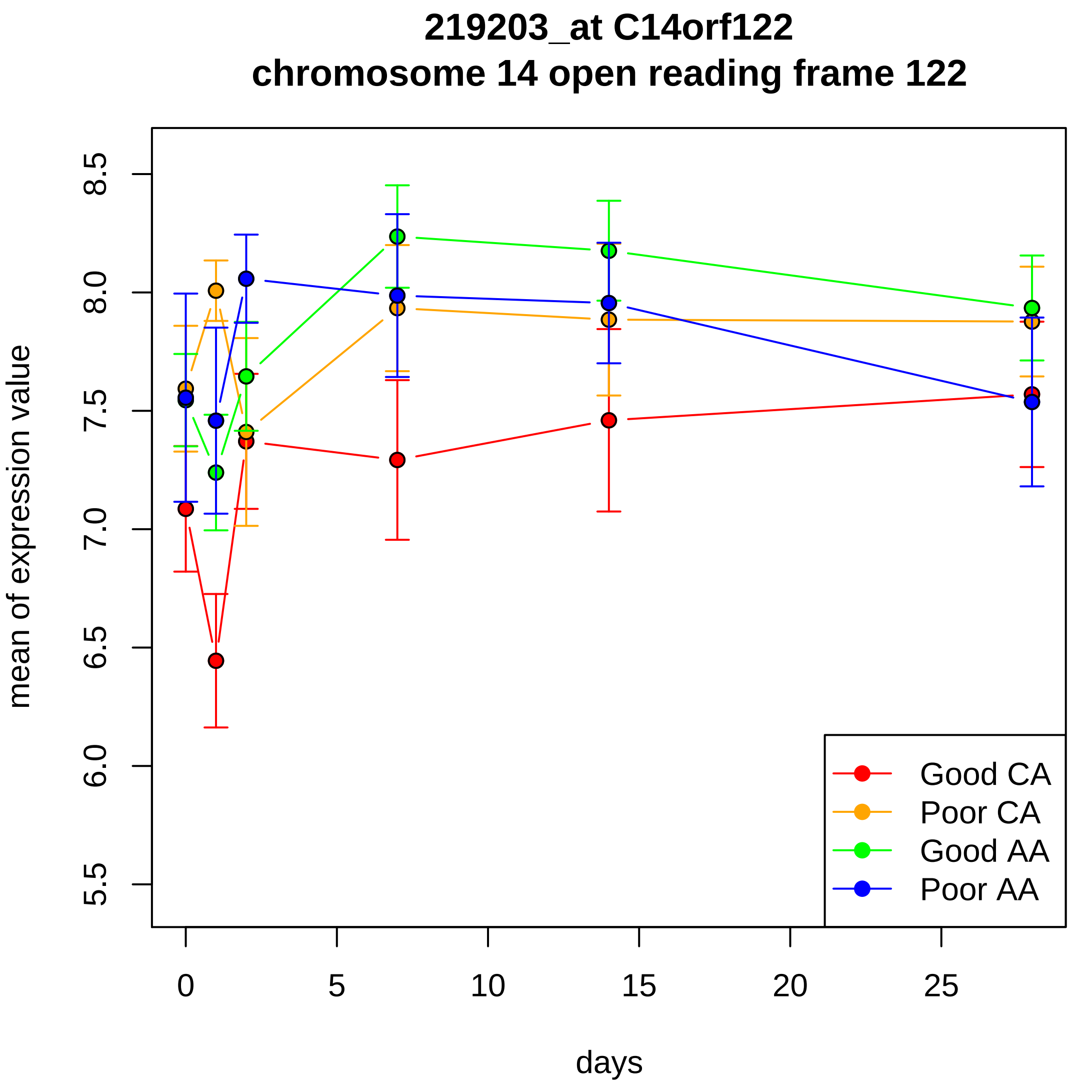
219203_at C14orf122
chromosome 14 open reading frame 122
mean of expression value
8.5
8.0
7.5
7.0
6.5
6.0
5.5
0
5
10
15
20
25
days
Good CA
Poor CA
Good AA
Poor AA

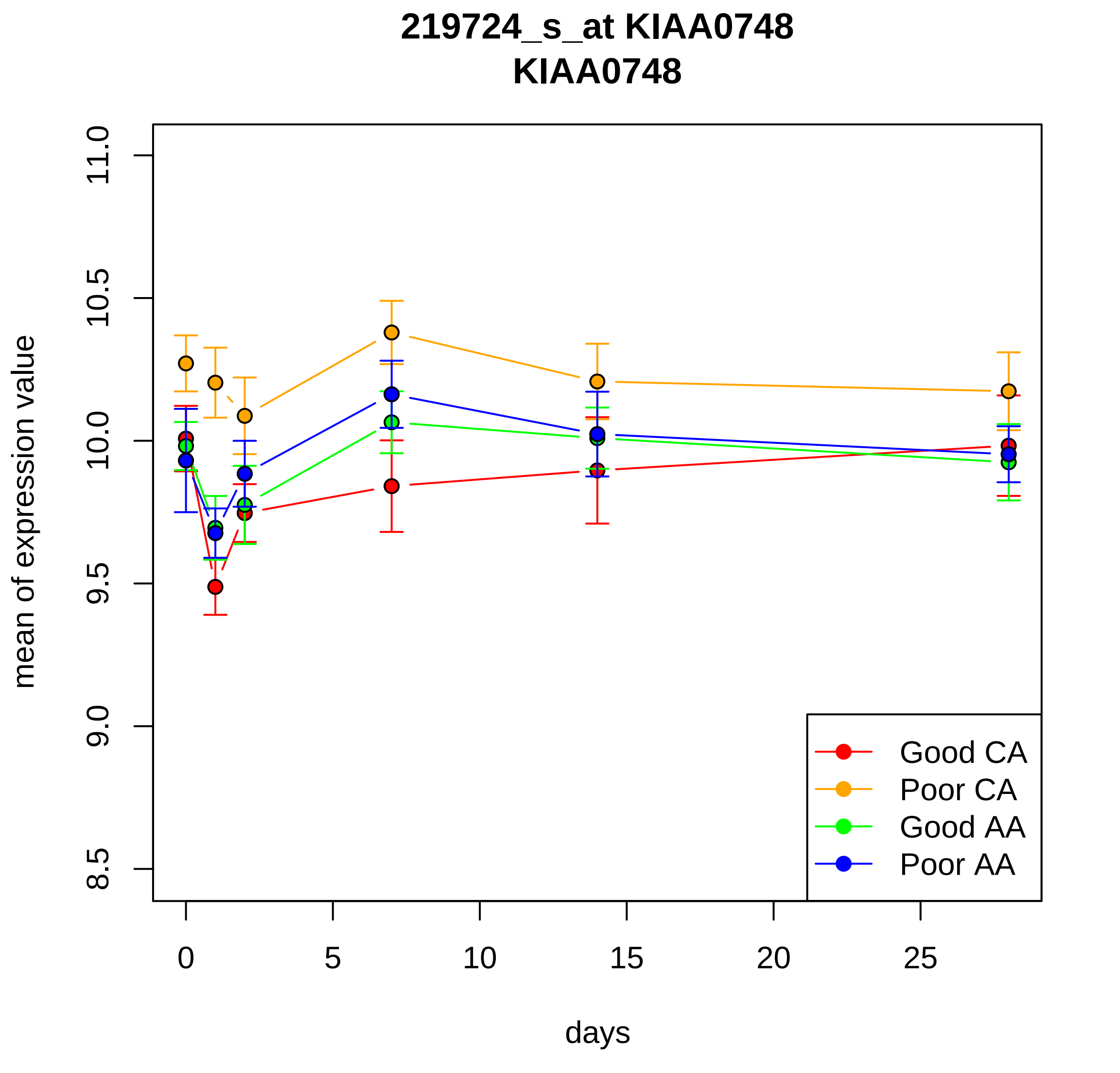
219724_s_at KIAA0748
KIAA0748
mean of expression value
days
8.5
9.0
9.5
10.0
10.5
11.0
0
5
10
15
20
25
Good CA
Poor CA
Good AA
Poor AA

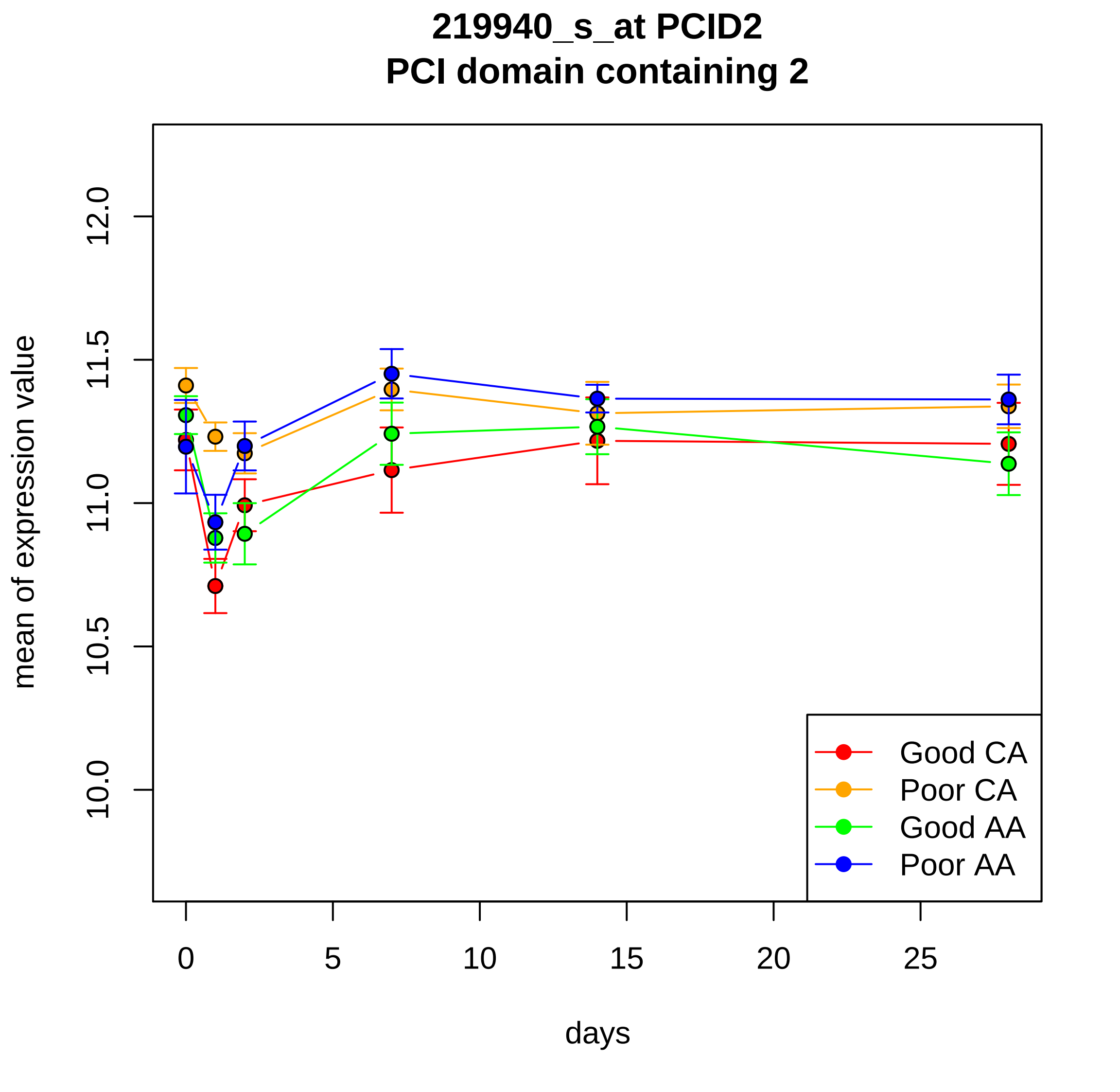
219940_s_at PCID2
PCI domain containing 2
mean of expression value
12.0
11.5
11.0
10.5
10.0
0
5
10
15
20
25
days
Good CA
Poor CA
Good AA
Poor AA

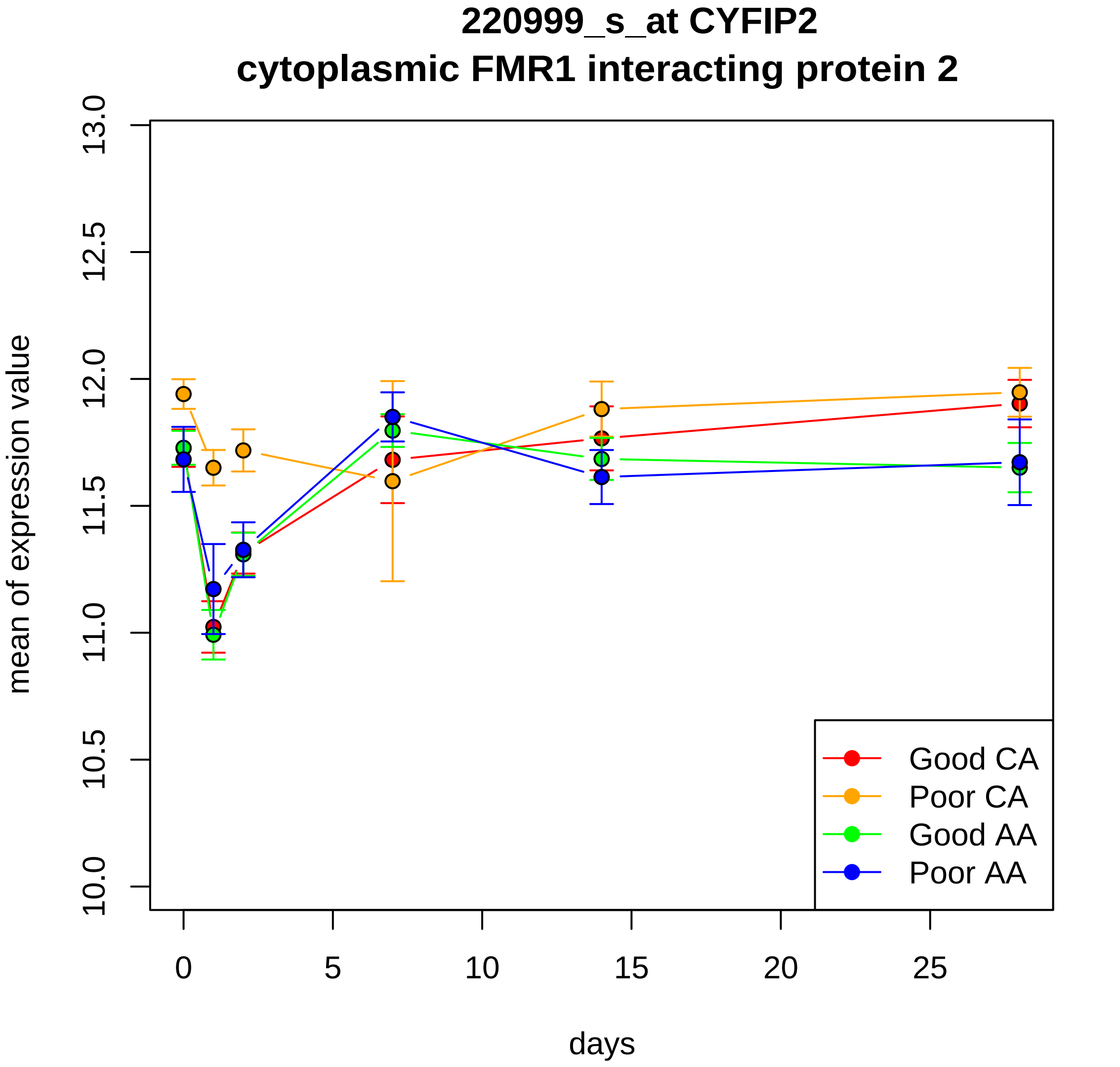
220999_s_at CYFIP2
cytoplasmic FMR1 interacting protein 2
mean of expression value
days
13.0
12.5
12.0
11.5
11.0
10.5
10.0
0
5
10
15
20
25
Good CA
Poor CA
Good AA
Poor AA

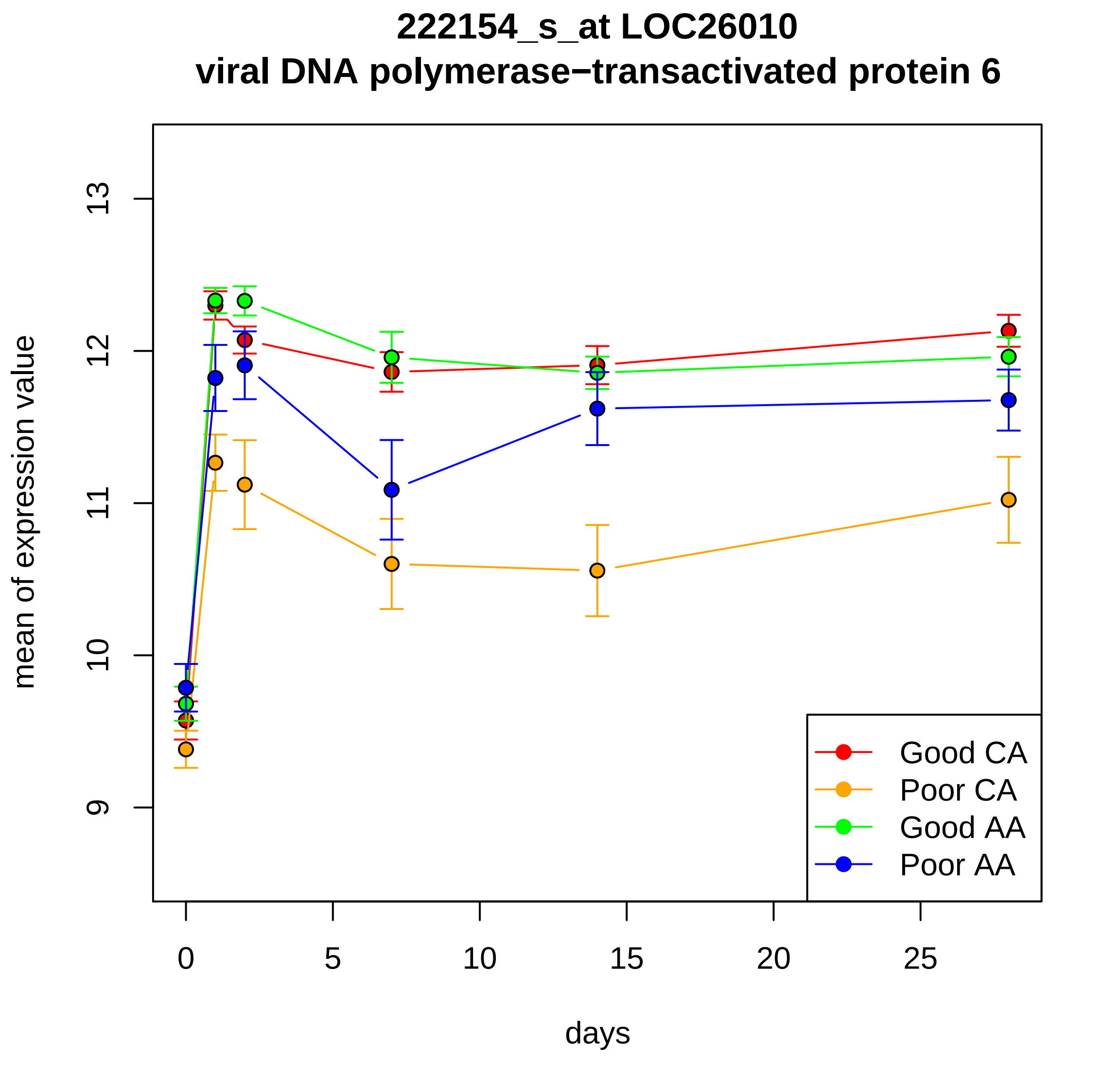

222154_s_at LOC26010
viral DNA polymerase−transactivated protein 6
mean of expression value
days
9
10
11
12
13
0
5
10
15
20
25
Good CA
Poor CA
Good AA
Poor AA

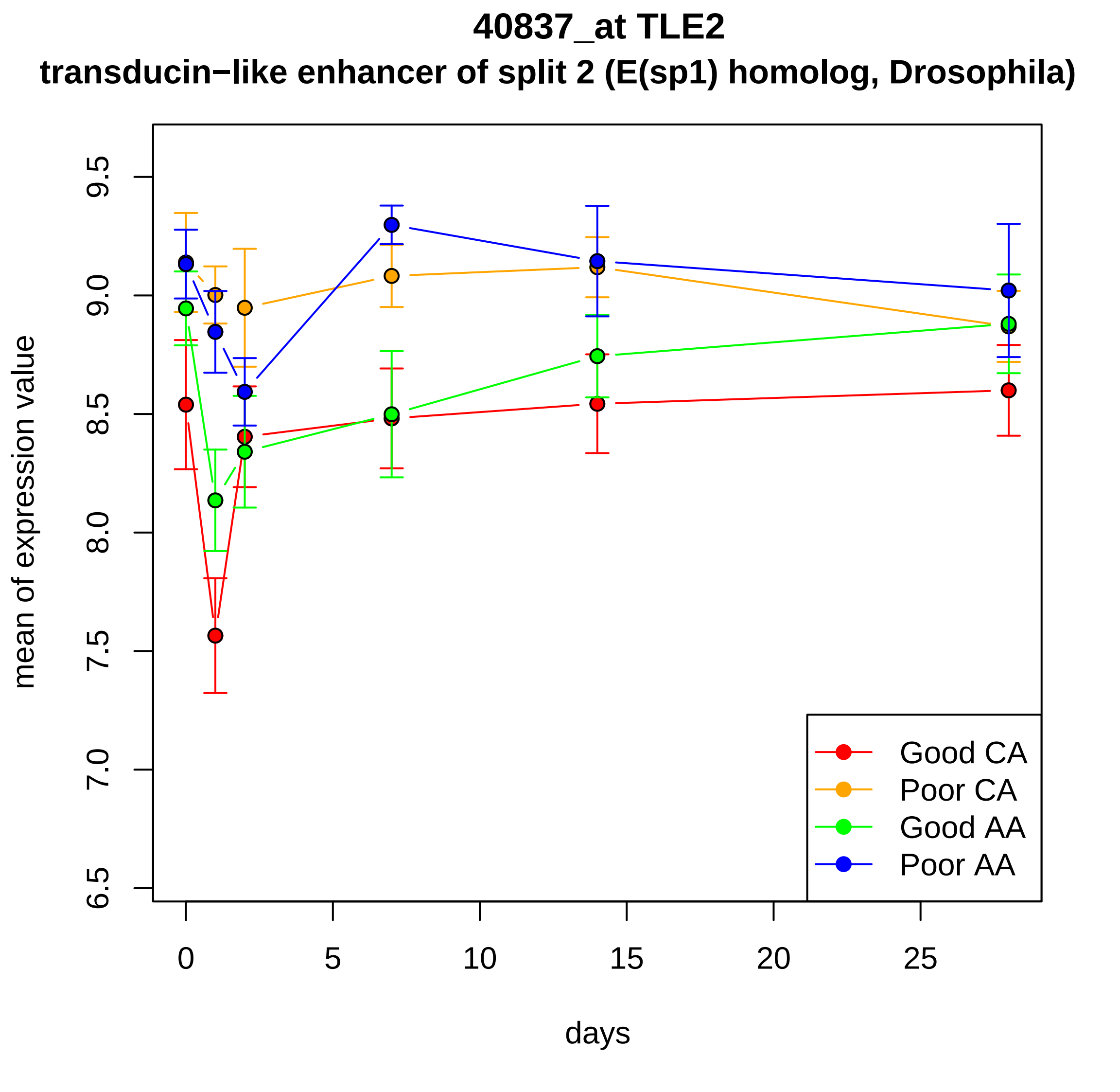

40837_at TLE2
transducin−like enhancer of split 2 (E(sp1) homolog, Drosophila)
mean of expression value
6.5
7.0
7.5
8.0
8.5
9.0
9.5
0
5
10
15
20
25
days
Good CA
Poor CA
Good AA
Poor AA
