# Supplementary figures and images for: The prediction of interferon treatment effects based on time series microarray gene expression profiles
Source: J Transl Med. 2008 Aug 9;6:44. doi: 10.1186/1479-5876-6-44 (PMC2546378; doi:10.1186/1479-5876-6-44)

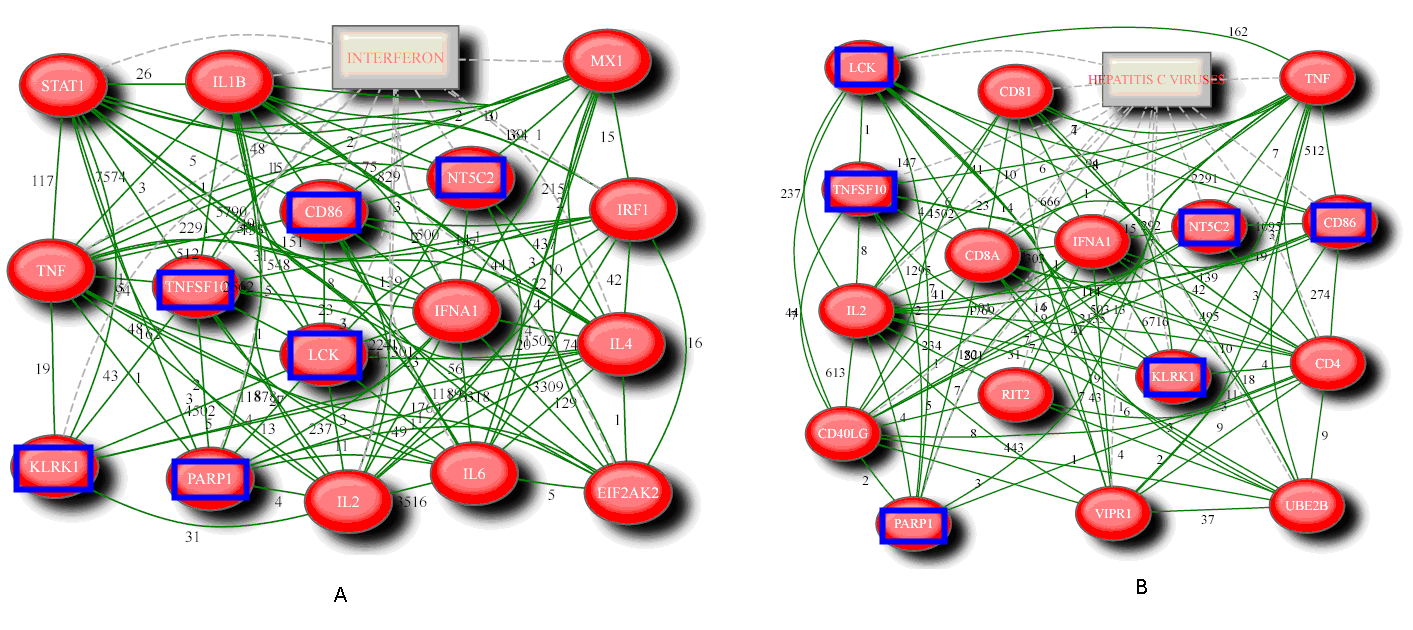

Supplement: Additional file 5 — Literature networks of thirty candidate biomarkers in relation to IFN (Interferon)/HCV (Hepatitis C viruses). The six genes that have direct connections with both IFN and HCV are framed with blue boxes. [file 1479-5876-6-44-S5.png]
